# Dissecting Efficacy and Metabolic Characteristic Mechanism of *Taxifolin* on Renal Fibrosis by Multivariate Approach and Ultra-Performance Liquid Chromatography Coupled With Mass Spectrometry-Based Metabolomics Strategy

**DOI:** 10.3389/fphar.2020.608511

**Published:** 2021-01-14

**Authors:** Lei Ren, Hao-Nan Guo, Jun Yang, Xiao-Ying Guo, Ye-Sheng Wei, Zhao Yang

**Affiliations:** ^1^ Department of Clinical Laboratory, Affiliated Hospital of Guilin Medical University, Guangxi, China; ^2^ Department of Clinical Laboratory, Daqing Oilfield General Hospital, Daqing, China

**Keywords:** metabolomics, UPLC-Q-TOF/MS, biomarker, metabolic pathway, natural product, renal fibrosis

## Abstract

Taxifolin (TFN) is an important natural compound with antifibrotic activity; however, its pharmacological mechanism is not clear. In this study, our aim is to gain insight into the effects of TFN and its potential mechanisms in unilateral ureteral obstruction (UUO) animal model using metabolomics approach to identify the metabolic biomarkers and perturbed pathways. Serum metabolomics analysis by UPLC-Q-TOF/MS was carried out to discover the changes in the metabolic profile. It showed that TFN has a significant protective effect on UUO-induced renal fibrosis and a total of 32 potential biomarkers were identified and related to RF progression. Of note, 27 biomarkers were regulated by TFN treatment, which participate in eight metabolic pathways, including phenylalanine, tyrosine and tryptophan biosynthesis, and phenylalanine metabolism. It also showed that metabolomics was a promising strategy to better dissect metabolic characteristics and pharmacological mechanisms of natural compounds by multivariate approach and ultra-performance liquid chromatography coupled with mass spectrometry.

## Introduction

Renal fibrosis (RF) is a common pathological characteristic of chronic kidney disease (CKD), which leads to final-stage renal disease (Hu et al., 2018; Yin et al., 2018; Kakitapalli et al., 2020; Rayego-Mateos and Valdivielso, 2020; Zeeh, 2020). It is generally recognized that RF is caused by activating various pathogenic factors such as inflammation reaction, injury, and drug stimulation (Zeisberg et al., 2002; Qin et al., 2011; Zhao et al., 2019). Then, the fibrogenic factors such as cytokines, growth factors, and chemotactic adhesion factors are released (Zeisberg and Kalluri, 2004; Liu, 2011; Vasko, 2016; Zhou et al., 2020). Up till now, renal biopsies and conventional biochemical detection are commonly applied to appraise the degree of RF. However, they are invasive, of high cost, and unstable and even have severe side-effects, which make accurate and repeated monitoring difficult in patients in the early stage (Jenkins et al., 2011; Chen et al., 2019). The common clinical treatment for RF, that is, to dilate the renal artery to increase the systemic blood perfusion, improve microcirculation disorder to enhance metabolism, alleviate the disorder of internal environment caused by hypoxia, and reduce toxic symptoms, is not ideal for patients (Boor et al., 2010; Zhang et al., 2018; Rayego-Mateos and Valdivielso, 2020).

Natural herbs characterized by multiple components, targets, and pathways have been widely utilized to cure various diseases for thousands of years (Li et al., 2020). Antifibrotic natural medicines with unique advantages have gained more and more attention for the treatment of RF (Shen et al., 2019; Sachan et al., 2020). Taxifolin (TFN), also called 3,5,7,3,4-pentahydroxy flavanone or dihydroquercetin, is a well-known natural flavonoid ingredient abundant in the Pinaceae tree family (Supplementary Figure S1) (Slimestad et al., 2007; Schauss et al., 2015; Topal et al., 2016). It possesses a wide range of biochemical and pharmacological properties in the management of oxidative stress, inflammation, tumors, microbial infections, and cardiovascular and liver disorders (Oi et al., 2012; Sun et al., 2014; Guo et al., 2015; Manigandan et al., 2015; Zhao et al., 2015; Gocer et al., 2016; Inoue et al., 2019; Zheng et al., 2019). Previous studies have proved that TFN exerts significant antioxidant effects that weaken cerebral ischemia-reperfusion injury by restraining oxidative enzymes and reactive oxygen species (ROS) production (Vladimirov et al., 2009; Voulgari et al., 2010). It enhances capillary microcirculation and antiplatelet aggregation and decreases the dose-dependent production of lipid-free radicals. In the transverse aortic constriction-induced animal model, TFN eliminates the phosphorylation of Smad2 and Smad2/3 nuclear translocation and restrains the superfluous production of ROS, ERK1/2, and JNK1/2 to weaken left ventricular fibrosis and collagen synthesis (Guo et al., 2015). It also suppresses the cholesterol esterification, triacylglycerol and phospholipid synthesis, apolipoprotein B secretion, and microsomal triglyceride synthesis in liver cells (Casaschi et al., 2004). Recent studies revealed that TFN protects against RF by upregulating the intracellular Nrf2 level and promoting nuclear translocation of Nrf2, regulating redox metabolites, and preventing TGF-β1-induced fibroblast activation and collagen synthesis (Wang et al., 2020). In addition, it cut back the concentrations of blood uric acid and creatinine and recovered the levels of caveolin-1/NF-κB signaling-related mRNA and proteins in diabetic nephropathy (Zhao et al., 2018). Unfortunately, the pharmacodynamics effect and potential molecule of TFN on RF have not been fully known owing to the limited scientific research data.

Currently, metabolomics based on high throughput and multivariate statistical analysis is generally applied in early diagnosis to discover the pathways associated with disease processes and drug treatment, disease classification, and prognosis (Zhang et al., 2013a; Zhang et al., 2013b; Zhang et al., 2013c; Wang et al., 2013; Zhang et al., 2014a; Zhang et al., 2014b; Wang et al., 2016; Li et al., 2017; Xie et al., 2017; Zhao et al., 2017; Sun et al., 2018a; Sun et al., 2018b; Li et al., 2018; Xie et al., 2019). Due to limited sensitivity and high data complexity, clear identification is usually limited to less than 100 metabolites (In et al., 2019; Zia et al., 2019). Mass spectrometry (MS) combined with triple quadrupole instruments show exceptional sensitivity and specificity for the measurement of approximately 1,000 metabolite peaks. However, it is necessary to know the precursor ion and the product ion of each metabolite in advance (Sun et al., 2012; Zhang et al., 2016a; Zhang et al., 2017; Fang et al., 2019; Tokuoka et al., 2019). To date, most kidney metabolomics studies have applied NMR- or MS-based methods. For example, a GC/MS-based metabolomics found that the urine alkane-alpha,omega-diamine and alpha,omega-dicarboxylic acid were abnormally upregulated in UUO-induced RF rats primarily involved in amino acid and sugar metabolism (Fang et al., 2016). There is a powerful connection between renal tubule interstitial fibrosis and glycerophospholipid metabolism and L-carnitine metabolism in the development of chronic renal failure (CRF) using UPLC-QTOF/HDMS-based plasma lipidomic and metabolomic approaches, and rhubarb extracts ameliorate glycerophospholipid, fatty acid, and amino acid metabolisms in adenine-induced chronic tubule interstitial nephropathy animal (Zhang et al., 2016b). In the present study, we emphatically explored the anti-RF efficacy and the underlying mechanisms of TFN by metabolomics strategy based on UPLC-Q-TOF/MS to identify the perturbed metabolic biomarkers and pathways changes.

## Materials and Methods

### Reagents

UPLC-MS grade methanol and acetonitrile were purchased from Merck Corporation (Merck, Germany). Formic acid was bought from Fisher Chemical Company (Geel, Belgium). Deionized water was purchased from the A.S. Watson Group, Ltd. (Hong Kong, China). Leucine-enkephalin with a purity of 99.10% was obtained from Sigma-Aldrich (St. Louis, MO, United States). Chloral hydrate and losartan were purchased from Shanghai Macklin Biochemical Co., Ltd. (Shanghai, China). TFN with a purity of 99.8% was provided by Nanjing Zelang Pharmaceutical Technology Co., Ltd. (Nanjing, China). The HPLC chromatographic conditions for TFN were demonstrated in Supplementary Figure S2. Interleukin-1β (IL-1β), tumor necrosis factor-α (TNF-α), superoxide dismutase (SOD), and malondialdehyde (MDA) enzyme-linked immunosorbent assay (ELISA) kits were bought from Jiancheng Bioengineering Institute (Nanjing, China). Serum creatinine (SCr) and blood urea nitrogen (BUN) ELISA kits were purchased from Longton Co., Ltd. (Shanghai, China). The antibody of transforming growth factor-β1 (TGF-β1) and small mothers against decapentaplegic (Smad-2) were purchased from Abcam (Cambridge, MA, United States) and used for immunohistochemical staining. The antibody of α-smooth muscle actin (α-SMA) and connective tissue growth factor (CTGF) were obtained from Beijing Laiyao Biological Technology Co., Ltd. (Beijing, China), and Beijing Century Aoke Biological Technology Co., Ltd. (Beijing, China), respectively. Primary antibodies against collagen type I, nuclear factor-kappaB (NF-KB), and fibronectin (FN) were produced by Cell Signaling Technology (Danvers, United States).

### Ethics

Fifty male SD rats in specific pathogen-free- (SPF-) grade (8 weeks old, weighing 180–200 g) were obtained by the Laboratory Animal Center of Guilin Medical University. After one week of adaptive feeding for animals under temperature of 24 ± 1°C, humidity of 55 ± 10%, and 12 h light/dark cycle with free access to standard chow and water, all the rats were randomized and divided into five groups (n = 10/group): sham operation group (control group), UUO rats group (model group), UUO rats treated with losartan group (UUO + LS), UUO rats treated with a high dose of TFN (UUO + TFN high), and UUO rats treated with a low dose of TFN (UUO + TFN low). All experimental procedures and animal care measures were executed in the light of the Guide for the Care and Use of Guilin Medical University.

### Animal Model

UUO model establishment was performed as described in the literature (Kakitapalli et al., 2020; Zeeh, 2020). Then, they were placed on the test bench in the supine position and shaved locally. Skin disinfection was applied on the middle of the abdomen, and the kidney and left ureter were exposed and separated using blunt dissection after opening the abdomen. Nipping with a hemostat at the upper middle section, the left ureter was ligated twice using 4-0 silk thread at the ends and then cut and eliminated between the two ligatures. The incision was cleaned and closed by a suture layer. The surgical method for rats in the control group was the same as that of the operation group, in which the abdominal cavity was separated, but no tissue was ligated or cut. The UUO-operated rats were injected with penicillin into the muscle three times. UUO model establishment was performed as described in the literature (Kakitapalli et al., 2020; Zeeh, 2020). Rats in every group received an intraperitoneal injection of 5% chloral hydrate (0.35 ml/100 g). Then, they were placed on the test bench in the supine position and shaved locally. Skin disinfection was applied on the middle of the abdomen, kidney, and ureter.

### Treatment

Before the surgery, UUO + TFN low and UUO + TFN high rat groups were given 8 and 16 mg·kg^−1^, respectively, by oral administration. Losartan was given by oral gavage at 10 mg/kg-1 every day for rats in UUO + LS group. Rats in the control and model group received sterile saline solution in the same volume and way. The administration duration of the drug was set to twenty-eight days. At the same time, other experimental operations and animal care procedures were carried out.

### Biochemical Indexes Detection

After TFN treatment for four weeks, rats in all groups were anesthetized with 10% chloral hydrate (4.0 ml/kg) on the 29th day. Blood samples were collected from the aorta abdominalis and transferred into the tube with heparin sodium. Blood samples were subsequently centrifuged at 3,500 rpm for 15 min at 4°C and the upper serum was collected for IL-1β, TNF-α, SOD, MDA, Scr, and BUN analysis. Olympus AU640 automatic biochemical analyzer was applied to disclose Scr and BUN levels. The blood of IL-1β, TNF-α, SOD, and MDA content was detected by ELISA according to the instruction in the kits.

### UPLC-Q-TOF/MS Analysis

A Waters ACQUITY™ ultra-performance liquid chromatography system (Waters Corp., Milford, United States) equipped with a Waters Synapt™ Q-TOF Mass system (Waters Corporation) was used for UPLC-Q-TOF/MS analysis. For UPLC-Q-TOF/MS detection, the serum needs to be further processed in addition to the above operations. Methanol in an ice-cold state was added to the serum sample to protein removal in the proportion of 3:1; then the mixture was centrifuged at 10000 rpm for 10 min at 4°C after mingling for 1 min using a mixer mill (Retsch GmbH & Co., Haan, Germany). The obtained liquid supernatant was dried under nitrogen and reconstituted with 200 μL methyl alcohol. Quality control samples (QCs) were prepared using a mixture of 10 μL plasma samples obtained from each sample in order to examine the stability of the instrument and optimize the analytical method. A QC filter was used to select and get rid of any ions with a coefficient of variation >15% during the analysis run.

### Histological Examination

Renal tissues were immediately harvested and superficial connective tissue was removed after washing with physiological saline. At least eight randomly histologic sections of the kidney in each group were chosen and stained with hematoxylin-eosin (H&E) for histological assessment of UUO model rats before and after TFN treatment. Renal tissues were fixed in 4% formaldehyde for 24 h, dehydrated with gradient alcohol, processed with xylene, paraffin-embedded, and sectioned at 5 µm thickness. The slices were baked and orderly placed in xylene dewaxing, underwent gradient alcohol dehydration, hematoxylin staining at 60°C for 10 min, 0.5% ammonia for 30 s, eosin staining for 10 min, and immersed in 80, 90, 95, and 100% alcohol for 1 min and transparent xylene for 5 min. Then, all the operated samples were observed under an optical microscope.

### Western Blotting

After thawing the kidney tissue preserved in liquid nitrogen, 100 mg kidney tissue was taken and added with a dissolution buffer containing protease inhibitor and benzamidepolyfluoride for homogenization. The supernatant was obtained as the total protein extract of kidney tissue in light of the instructions of the protein concentration detection kit. The obtained proteins underwent SDS-PAGE electrophoresis separation in the conventional operation method and then transferred to PVDF membrane. After incubating 5% nonfat milk powder at room temperature for 2 h, the protein samples were added to primary antibody working solution, reacted at 4°C for 24 h, and washed 5 times at 10 min/time. Then, a secondary antibody working solution was addedto the sample and incubated for 1 h at room temperature; the membrane was washed 5 times with TBS-T at 10 min/time.

### Metabolomics Analysis

Detailed parameters of chromatographic separation and mass spectrometry were depicted as follows: the metabolomics profiling analysis was performed on a Waters BEH C18 (2.1 × 100 mm, 1.7 μm) using 0.05% (v/v) formic acid water solution as mobile phase A and 0.05% (v/v) formic acid acetonitrile solution as mobile phase B running with a gradient program of 5–30% B in 0–3.5 min, 30–65% B in 3.5–6 min, 65–85% B in 6–9 min, 85–95% in 9–10 min, 95% B in 9–11 min, and 2 min of balance back to 5% B; the flow rate was set constant at 0.4 ml/min and the column temperature was maintained at 35°C for all samples; the injection volume was set to 2 μL; under ESI+ and ESI- ion scanning mode, capillary voltages were, respectively, 3,500 and 3000 V; nebulizer pressure was controlled at 32 psi; nozzle voltage was set at 250 V; drying gas temperature was set to 350°C at 20 L/min; sheath gas was set to 400°C at 16 L/min. The acquisition mass range was from m/z 50 to m/z 1,500 in full-scan mode.

The precision and reproducibility were analyzed for evaluating the above-mentioned developed UPLC-Q-TOF/MS method. Raw data were processed using the Progenesis QI data analysis software (Version 2.0, Nonlinear Dynamics, Newcastle, United Kingdom), which permits deconvolution, alignment, and data reduction in order to export a list of mass, retention time, m/z, and corresponding intensities for all the detected peaks from each sample. The principal parameters were controlled as follows: retention time range, 1–11 min for metabolomic analysis; mass range, 50–1,500 m/z; mass tolerance, 0.01; minimum intensity, 1%; mass window, 0.05; retention time window, 0.20; noise elimination level, 6.

Multivariate analysis, such as principal component analysis (PCA), orthogonal partial least squares discriminant analysis (OPLS-DA), and variable weight value (VIP) plot, was carried out using SIMCA software (version 14.0, Umetrics AB, Umeå, Sweden). PCA score plot of spectral data has the ability to visualize overall clustering, appraise the main sources of variation, and remove outliers beyond the confidence interval (95%). For the purpose of filtering endogenous metabolites that play a vital role in metabolic profile, the OPLS-DA model performed one hundred permutation validations to assess the fitting of the discriminant analysis.

In the VIP scatter plot that highlight the variable weight value of the relevant reactive ion contribution degree, the ion fragments from the bottom with small VIP value have a smaller contribution to differential metabolism. On the contrary, the top ions in V-shape with larger VIP value provide more contribution to the metabolic profile trajectory difference among groups. Meanwhile, the ions that keep away from the original point in the loading plot obtained from OPLS-DA analysis seem as potential metabolites. Differential metabolites meet the conditions as follows: VIP value more than 1.5 and calculated *p* value in both Student’s *t*-test and Mann–Whitney test less than 0.05.

The identification of metabolites was firstly performed to retrieve by available biochemical databases, such as HMDB, KEGG, and Chemspider, and subsequently was verified by MS data, MSE fragments, molecular weights, and chemical element compositions. The topological trait of metabolic pathways related to the RF model and anti-RF efficacy of TFN was described by analysis software (MetPA) in MetaboAnalyst 4.0 combined with various advanced path analysis programs to highlight the role of perturbed metabolic biomarkers and pathways in the biological system.

All the acquired data were confirmed by the Kolmogorov–Smirnov test and were found to meet the peculiarity of the normal distribution. The results of conventional efficacy evaluation and metabolomics analysis were carried out in SPSS (version 22.0, SPSS Inc., Chicago, IL, United States), which were expressed as mean ± standard deviation (SD). The differences of mean values among groups were tested by Student’s *t*-test or one-way ANOVA followed by Tukey’s *post hoc* test. *p* values <0.05 were deemed to be statistically significant, and *p* values <0.01 were deemed to be more statistically significant.

## Results

### Histological Changes Analysis

To appraise the changes of tissue fibrosis before and after TFN treatment, high-power field optical microscope was used for each section from different groups. Pathological results showed that the kidney tissue of the control group presented clear glomerular and renal tubular structures, and there was no inflammatory cell infiltration in the renal interstitium, no fibrous tissue hyperplasia, and no mesangial cell or stromal tissue hyperplasia (Supplementary Figure S3). In the model group, glomerular stromal tissue hyperplasia, tubular epithelial cell swelling, vacuolar degeneration, focal necrosis, flaky atrophy, and even epithelial cell necrosis and shedding can be seen in the tubular lumen. Renal interstitial inflammatory cells are diffusely infiltrated, leading to fibrous tissue proliferation and even sheet fibrosis. After 8 and 16 mg·kg^−1^ of TFN treatment, the UUO-related histopathologies were moderated, and the pathological results were similar to those of rats under sham operation. There were less inflammatory cell infiltration, neatly arranged renal tubules, and a small interstitial fibrosis area.

### Biochemical Indexes

**FIGURE 1:**
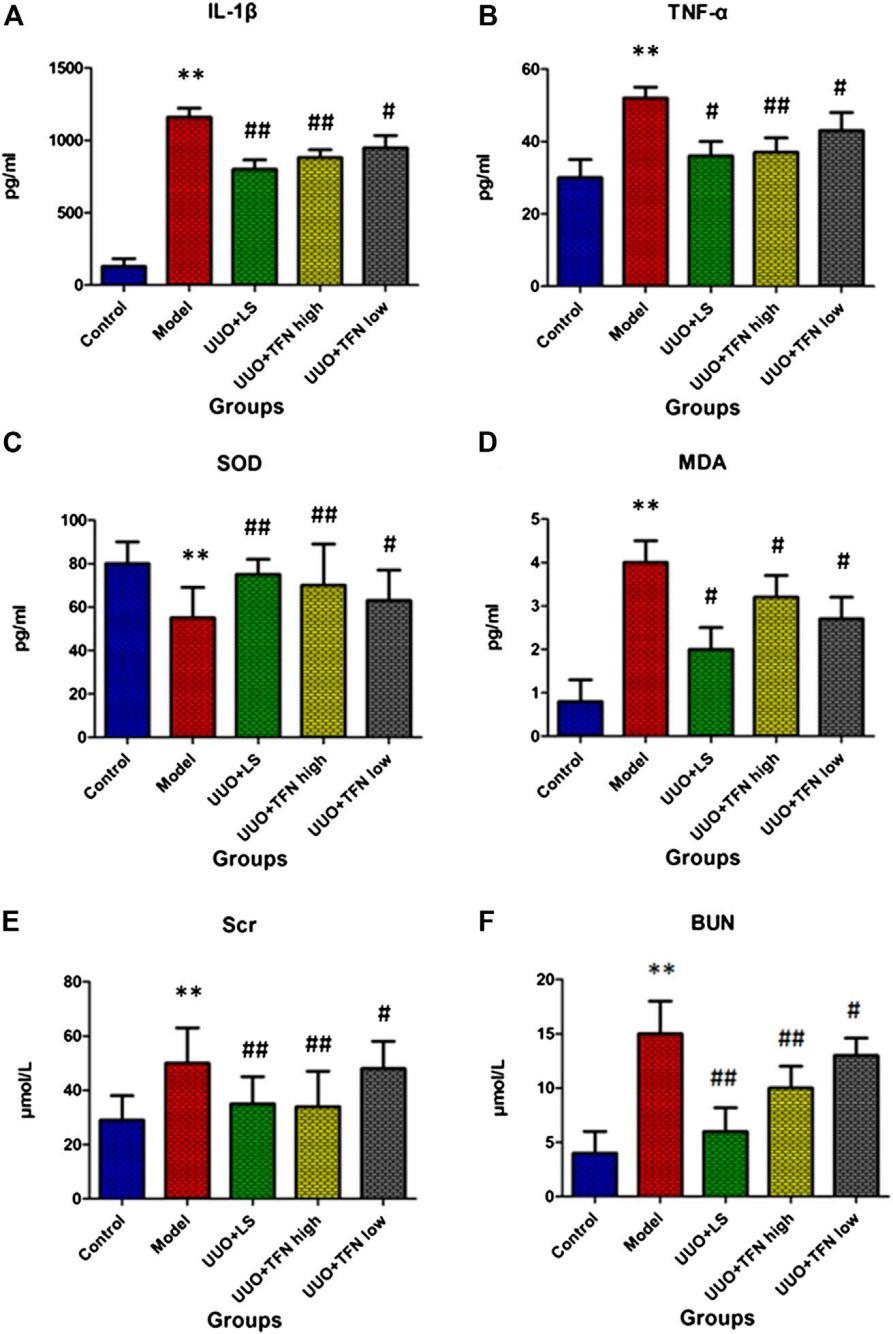
The changes of IL-1β, TNF-α, SOD, MDA, Scr, and BUN content in different groups after twenty-eight days of TFN administration. *Note.* The difference of mean values among groups was tested by Student’s *t*-test or one-way ANOVA followed by Tukey’s *post hoc* test. *Model group vs. control group, *p* < 0.05; **model group vs. control group, *p* < 0.01; #treament group vs. model group, *p* < 0.05; ##treament group vs. model group, *p* < 0.01, (n = 10/group).

The changes of IL-1β, TNF-α, SOD, MDA, Scr, and BUN content after twenty-eight days of TFN administration are shown in Figures 1A–F. Compared with the control group, inflammatory factors, including IL-1β and TNF-α, showed a significantly elevated value. Redox reaction indexes of SOD were significantly lower than those in the control group, and the MDA level was distinctly higher than that in the control group. Renal function indexes, including Scr and BUN, in the model group were significantly higher than those in the control group. Compared with the model group, TFN could decrease IL-1β, TNF-α, MDA, Scr, and BUN levels in a dose-dependent manner, whereas TFN could also significantly increase the SOD level. However, the low dose of TFN showed little effect on serum TNF-α level.

**FIGURE 2:**
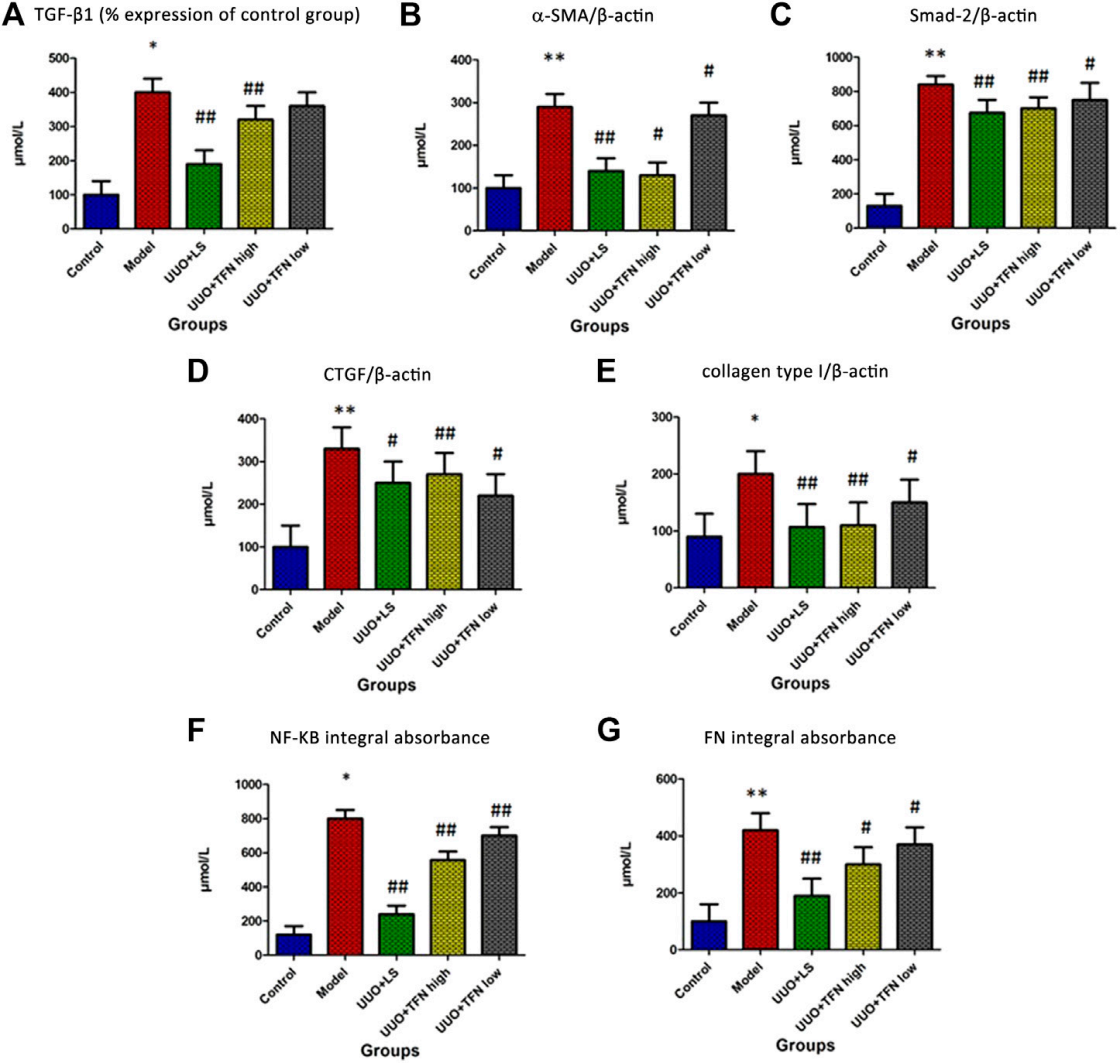
Immunohistochemical analysis of TGF-β1, Smad-2, α-SMA, CTGF, collagen type I, NF-KB, and FN content in kidney tissue. *Note.* The difference of mean values among groups were tested by Student’s *t*-test or one-way ANOVA followed by Tukey’s *post hoc* test. *Model group vs. control group, *p* < 0.05; **model group vs. control group, *p* < 0.01; # treament group vs. model group, *p* < 0.05; ##treament group vs. model group, *p* < 0.01, (n = 10/group).

As demonstrated in Figures 2A–G, TGF-β1, Smad-2, α-SMA, CTGF, collagen type I, NF-KB, and FN contents in kidney tissue were upregulated when compared with the normal renal tissues. Compared with the model group, TFN could decrease TGF-β1, Smad-2, α-SMA, CTGF, collagen type I, NF-KB, and FN levels in a dose-dependent manner.

### Metabolic Profile Changes

**FIGURE 3:**
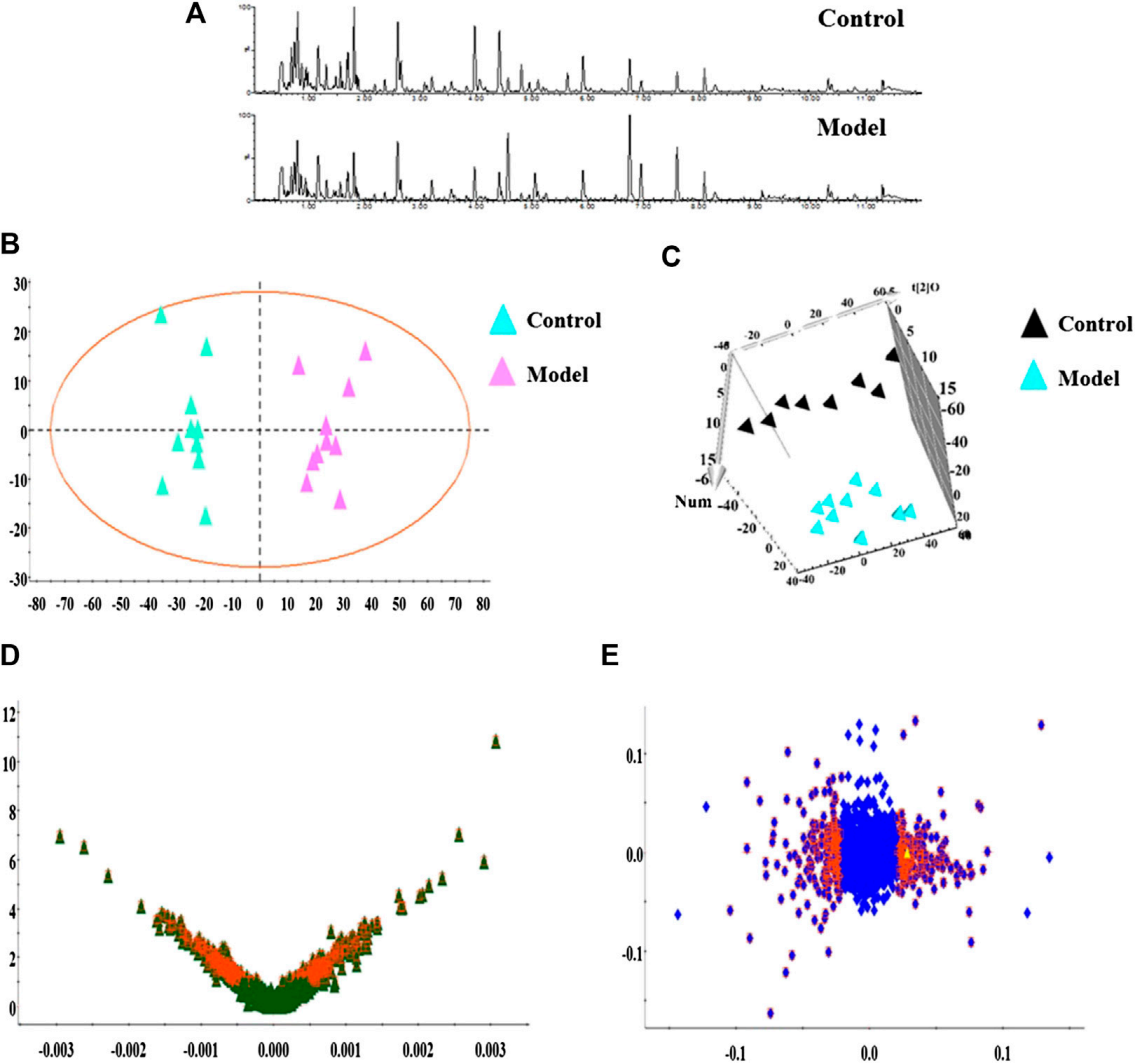
Metabolomics profiling of the control group and model group in positive mode. Note: **(A)** typical BPI metabolic profiles; **(B)** 2D PCA score plots; **(C)** 3D OPLS-DA score plot; **(D)** VIP plot of serum profile scanned by OPLS-DA analysis; **(E)** loading plot of serum profile scanned by OPLS-DA analysis.

**FIGURE 4:**
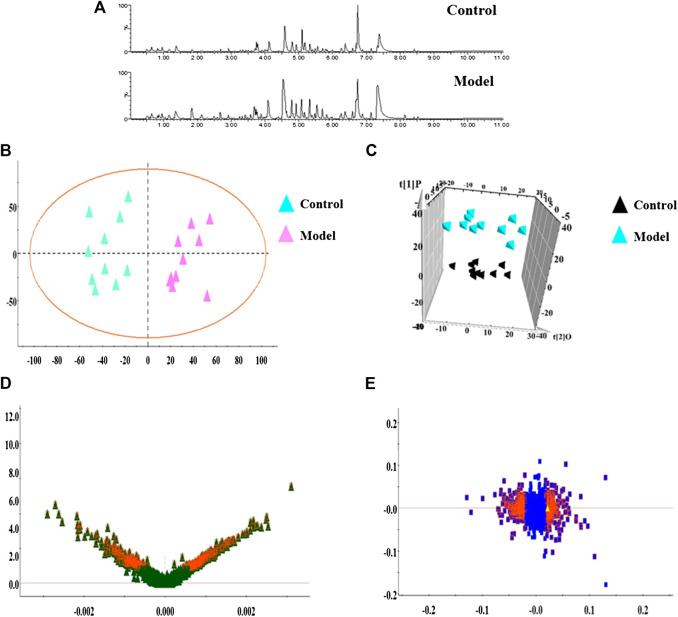
Metabolomics profiling of the control group and model group in negative mode. Note: **(A)** typical BPI metabolic profiles; **(B)** 2D PCA score plots; **(C)** 3D OPLS-DA score plot; **(D)** VIP plot of serum profile scanned by OPLS-DA analysis; **(E)** loading plot of serum profile scanned by OPLS-DA analysis.

In this research, serum samples from five groups were searched in ESI+ and ESI− ion mode under the UPLC-Q-TOF/MS system, in which typical BPI metabolic profiles of control and model group in Figures 3A,4A were obtained by SIMCA V14.0 software. It was not hard to see that metabolic profiling of serum is similar and there were only distinct content differences in mapping. For further seeking endogenous differentiated metabolites to assess UUO-induced RF animal model and TFN efficacy, a nontargeted metabolomics strategy was carried out for the multivariate data analysis. 2D PCA score plots of both ion modes were shown in Figures 3B,4B, in which each spot represented a sample, and the control groups were clearly clustered and separated to the model group, indicating that the RF rat model has been successfully established at serum metabolism level. From Figures 3C,4C, the parameters R2X, R2Y, and Q2 obtained by cross-validation were, respectively, 0.923, 0.8996, and 0.642 in positive mode, and those parameters were, respectively, 0.931, 0.908, and 0.710 in negative mode, indicating the data model possesses good prediction ability and reliability. In addition, the 2D OPLS-DA score plot showed a notable separation. The loading plot (Figures 3E,4E) and VIP plot diagrams of OPLS-DA (Figures 3D,4D) were generated to know the contribution rate between different groups. The further away they were from the origin, the greater the contribution was to the clustering of the control group and model group.

Potential endogenous biomarkers that meet the condition of *p* < 0.05 and VIP value more than one were firstly screened by Student’s *t*-test and Mann–Whitney test. Then, the precise molecular weight within a reasonable measurement error range, element composition, and unsaturation and structure were determined according to Rt, accurate quality, MS/MS data from the UPLC-MS platform, and online databases such as HMDB and KEGG. A total of 32 potential biomarkers were identified and characterized, in which the details of 15 in positive ion mode and 17 in negative ion mode were listed in Supplementary Table S1, including isocitric acid, ornithine, 3-hydroxyanthranilic acid, picolinic acid, citric acid, uric acid, asparagine, tryptophan, mevalonic acid-5P, glutamine, SM (d18:1/22:0), kynurenic acid, hydroxytyrosol, cyclic GMP, 20-hydroxyeicosatetraenoic acid, deoxyuridine, prostaglandin F2a, phenylalanine (PA), arachidonic acid, LysoPC (17:0), LysoPC (15:0), palmitoleic acid, SM (D18:0/16:1), galabiosylceramide, LysoPC (16:1 (9Z)), oleic acid and sphinganine, 5′-methylthioadenosine, cysteinylglycine, pregnenolone sulfate, dodecanoic acid, and dityrosine. Sixteen metabolic pathways with impact value more than zero were closely related to pathogenesis and development of model rats in serum metabolism level, including PA, tyrosine and tryptophan biosynthesis, PA metabolism, arachidonic acid metabolism, sphingolipid metabolism, terpenoid backbone biosynthesis, citrate cycle (TCA cycle), alanine, aspartate and glutamate metabolism, arginine and proline metabolism, arginine biosynthesis, pyrimidine metabolism, glutathione metabolism, tryptophan metabolism, glyoxylate and dicarboxylate metabolism, cysteine and methionine metabolism, purine metabolism, and glycerophospholipid metabolism, as shown in Supplementary Figure S4.

### TFN Effect on Perturbed Biomarker

**FIGURE 5:**
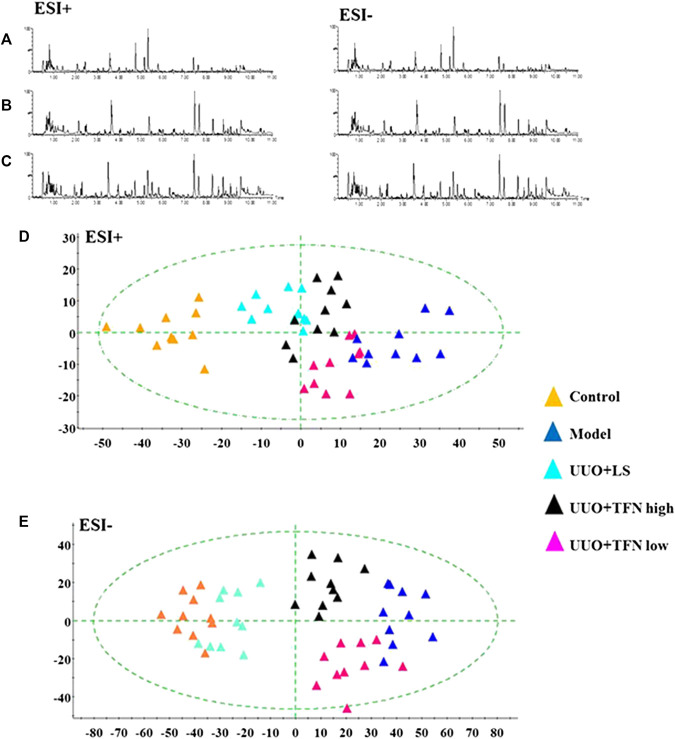
Metabolomics profiling of the control, model, UUO + LS, UUO + TFN high, and UUO + TFN low group in positive mode. **(A)** Typical BPI metabolic profiles of UUO + LS group in both ion mode; **(B)** typical BPI metabolic profiles of UUO + TFN low group in both ion modes; **(C)** typical BPI metabolic profiles of UUO + TFN high group in both ion modes; **(D)** 2D PCA score plots of five groups in positive mode; **(E)** 2D PCA score plots of five groups in negative mode.

**FIGURE 6:**
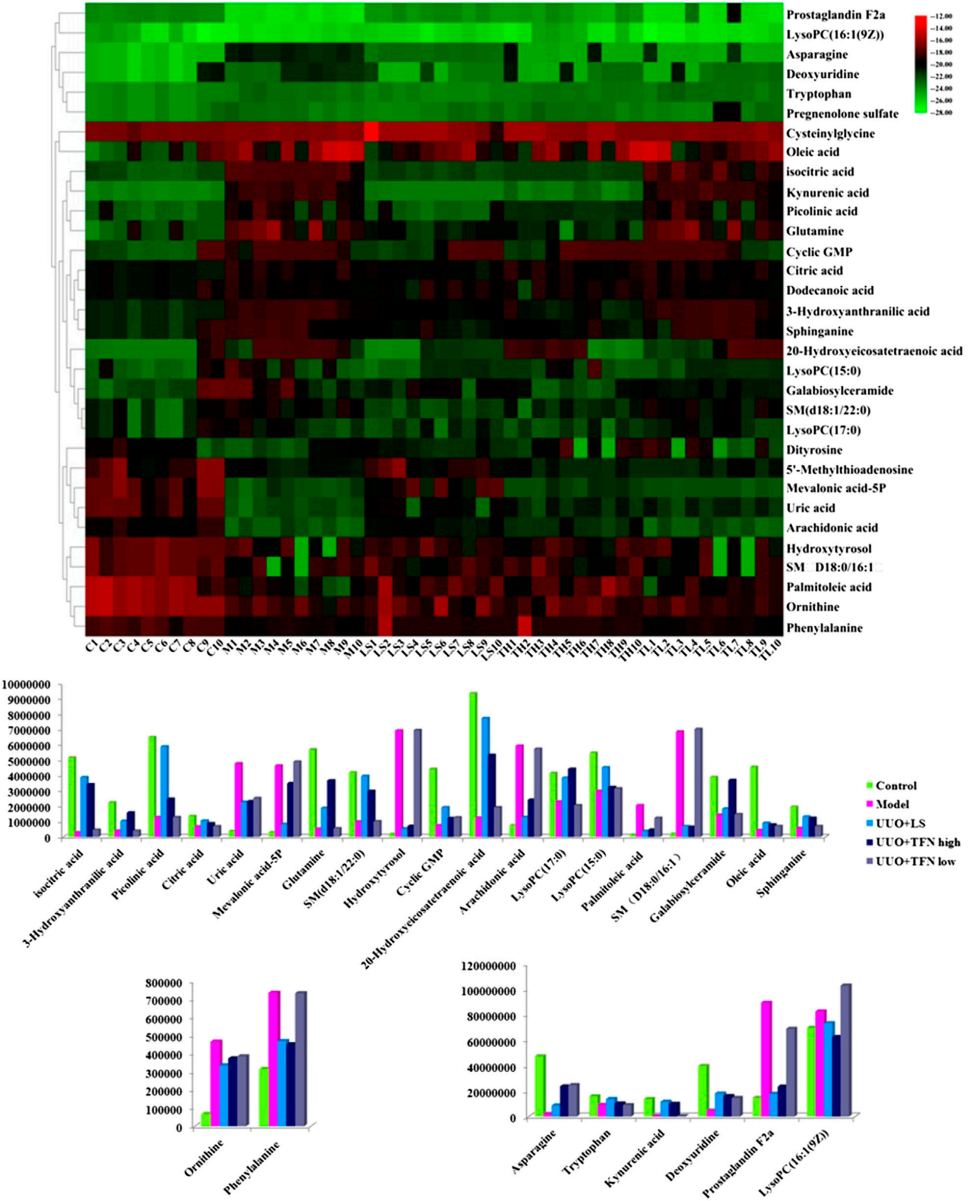
Heatmap of all the potential biomarkers regulated by TFN. Different color marks suggest the change degree of relative content, in which the darker the color, the higher the relative content.

From typical BPI metabolic profiles of UUO + TFN and UUO + LS group in Figures 5A–C, it was shown that the peaks in metabolic profiling exist in content differences. PCA was performed on the serum metabolic profile of five groups of rats, which were generally clustered together in an individual group with similarity, and a clear separation between groups was detected, suggesting that these five groups were differential. As shown in Figures 5D,6E, the UUO + TFN group and UUO + LS located between the control and model group present a similar changing trend, where UUO + TFN high group is closer to the control group than UUO + TFN low group, manifesting TFN plays a vital intervention role in reversing the metabolic profile of RF model animals to make them in a healthy state in dose-dependent manner. Compared with the model group, 17 metabolite level expressions in the UUO + TFN group were significantly increased, such as isocitric acid, 3-hydroxyanthranilic acid, picolinic acid, citric acid, asparagine, tryptophan, glutamine, SM (d18:1/22:0), kynurenic acid, cyclic GMP, 20-hydroxyeicosatetraenoic acid, deoxyuridine, LysoPC (17:0), LysoPC (15:0), galabiosylceramide, oleic acid, and sphinganine, whereas the levels of 10 metabolites were significantly decreased such as ornithine, uric acid, mevalonic acid-5P, hydroxytyrosol, prostaglandin F2a, PA, arachidonic acid, palmitoleic acid, SM (D18:0/16:1), and LysoPC (16:1 (9Z)). Among them, UUO + TFN low group can affect the content of 16 metabolites, whereas UUO + TFN high group can regulate 27 metabolites. In Figure 6A, the brightness difference of color in the heatmap reveals the relative content changes of 27 potential metabolites in five groups to highlight the TFN pharmacological activity during treatment. The relative peak areas of the above-mentioned metabolites were shown in a bar graph from Figure 6B.

**FIGURE 7:**
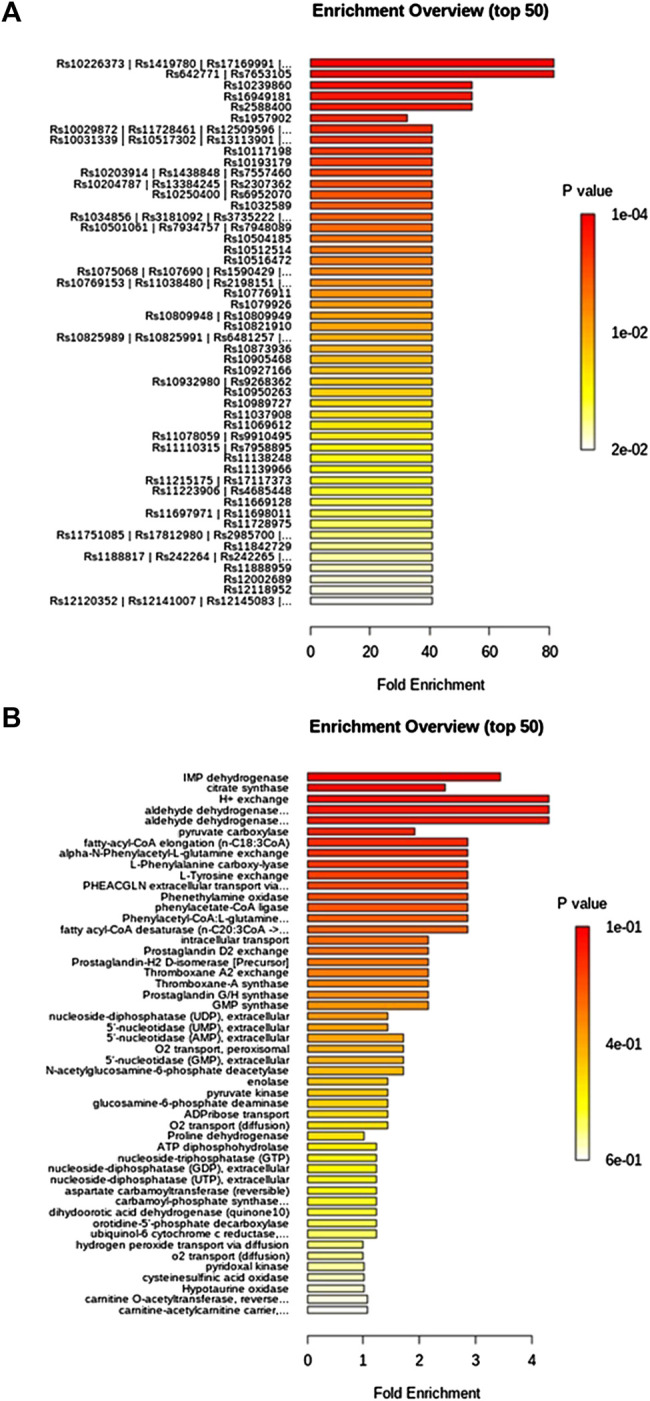
Single-nucleotide polymorphisms loci and dysfunctional enzymes were detected by genome-scale network model of human metabolism after TFN treatment on UUO-induced RF rats.

TFN in low dosage callback serum biomarkers mainly involved arachidonic acid metabolism, sphingolipid metabolism, citrate cycle (TCA cycle), alanine, aspartate and glutamate metabolism, and arginine and proline metabolism with impact value greater than 0.1 in Supplementary Figure S3A, and TFN in high dosage regulated serum biomarkers such as PA, tyrosine and tryptophan biosynthesis, PA metabolism, arachidonic acid metabolism, sphingolipid metabolism, terpenoid backbone biosynthesis, citrate cycle (TCA cycle), alanine, aspartate and glutamate metabolism, and arginine and proline metabolism with impact value greater than 0.1 in Supplementary Figure S3B. It was indicated that TFN plays a role in preventing and treating RF by interfering with the above metabolic pathways. The information of single-nucleotide polymorphisms (SNPs) loci and dysfunctional enzymes were detected by genome-scale network model of human metabolism in Figure 7.

## Discussion

Metabolomics research has begun to outline the changes in metabolites from blood and urine at different stages of nephropathy in order to provide insights into nephropathy at the molecular level. However, some current challenges limit the interpretation of modern research (Schrimpe-Rutledge et al., 2016). In particular, metabolomics cannot fully cover the cognition of metabolites on different platforms and there is a crisis of reproducibility in the study of clinical biomarkers (Casadei et al., 2018). These limitations may be resolved through continuous developments in MS sensitivity and mass accuracy and strength to add currently unknown m/z peak identities and standardize reagents and terminology (Gowda and Djukovic, 2014). The combined dataset will provide enhanced statistical capabilities for integrating metabolomics data with genomics and other functional genomics outputs (Telenti, 2018). In turn, these achievements will provide insight into the genetic determinants of selected metabolites alteration and whether metabolite markers of kidney disease have causality or association relationship with the emphasized pathways. The epidemiological scale should be combined with physiological and experimental research to provide more direct insights into organ specificity and the underlying mechanisms of certain metabolite changes (Wu et al., 2016; Lai et al., 2018).

The results of HE staining in this study showed that RF rats displayed glomerular hyperplasia in the glomeruli, swollen tubule epithelial cells, tubule vacuoles degeneration, necrosis, atrophy, and infiltration of renal interstitial inflammatory cells. In the control group, renal tubular epithelial cells and renal interstitium were generally normal. The above morphological studies are consistent with relevant literature reports (Chen and Li, 2018), indicating that the animal model replication method used in this experiment is feasible and successful. Administration of TFN for 4 weeks can improve the infiltration of inflammatory cells and the swelling of renal tubular epithelial cells and reduce the area of interstitial fibrosis. Tissue staining shows that the renal tubular structure is clear and there are no degeneration and necrotic cells in the lumen; in addition to the above changes, the epithelial cells of the tube wall are regularly arranged without atrophy, the renal interstitial structure is clear, and there is no fibrosis. It also shows that the antirenal fibrosis of TFN is mainly manifested in renal tubular epithelial cells. The study found that TNF-α can directly induce apoptosis of renal tubular epithelial cells and simultaneously upregulate the expression of IL-1β, which is consistent with the results of this experiment; that is, the content of TNF-α and IL-1β in the serum of the model group was increasing (Donnahoo et al., 1999). SOD that can block and resist the damage of oxygen free radicals to renal tubular epithelial cells and may repair damaged renal tubular epithelial cells is the most important substance in the body to scavenge oxygen free radicals. It is an intuitive way to observe cell damage and repair index (Son et al., 2008). Our results showed that TFN could effectively reduce the TNF-α and IL-1β activity and increase the SOD activity of rats caused by UUO for enhancing the antioxidant capacity, repairing damaged renal tubular epithelial cells to alleviate the process of renal interstitial fibrosis. MDA level can indirectly reflect the degree of damage to renal tubular epithelial cells and is a cytotoxic substance produced during the peroxidation reaction (Nomani et al., 2018). Scr and BUN are two important indicators for evaluating renal function, which both are excreted by the kidney. Creatinine is the end product of creatine metabolism in muscle tissue, and urea nitrogen is the end product of protein metabolism (Hosten, 1990). Scr and BUN reflect the ability of the kidney to clear creatinine from the blood and concentrate it in urine. This study found that the Scr and BUN contents of the model group were upregulated. Compared with the model group, MDA, Scr, and BUN levels were downregulated after 8 and 16 mg·kg^−1^ TFN in gavage way, indicating that TFN can ameliorate UUO-induced tubular epithelial damage. TGF-β 1 is a key renal interstitial fibrosis-promoting factor, and TGF-β 1 is one of the strongest known fibrotic cytokines (López-Hernández and López-Novoa, 2012). In the TGF-β 1 signal transduction pathway, the Smad-2 pathway is recognized as one of the most important downstream pathways and the only known intracellular kinase receptor substrate. TGF-β 1/Smad-2 signaling pathway is the core pathway of RF. Through experiments, compared with the control group, the expressions of TGF-β 1 and Smad-2 in the rat kidney were increased in the model group, indicating that the TGF-β1/Smad-2 signal transduction pathway is involved in UUO-induced renal interstitial fibrosis. TGF-β1 promotes renal interstitial fibrosis to destroy the basement membrane, then enhances the expression of α-SMA and other myofibroblast markers, and promotes the synthesis of collagen types I and III in the extracellular matrix (Wang et al., 2019). CTGF as a newly discovered fibrogenic factor can be produced by interstitial cells such as fibroblasts under the action of TGF-β1 and participates in the effect of TGF-β1 on interstitial cells to promote cell proliferation and extracellular matrix synthesis to accelerate the process of tissue fibrosis. In resting cells, NF-κB and IκB form a complex that does not play a role in the cytoplasm. When cells are stimulated by extracellular inflammatory signals, the inhibitor of NF-κB dimer k (IκBk) is inactivated, leading to phosphorylation of IκB, and the nuclear localization site of NF-κB is exposed. The rapid translocation of κB and specific IκB induces target gene transcription, promotes the proliferation of target cells, such as TNF-α and ICAM-1, and triggers renal damage (Oh et al., 2014; Suh et al., 2015). TGF-β1 can also induce NF-κB-mediated renal inflammation. FN, as a glycoprotein with a relative molecular mass of 250,000, interacts with a variety of matrix proteins and regulates a variety of cellular processes (Stevens et al., 2007; Xie et al., 2012). NF-κB activation can regulate the overexpression of adhesion molecules ICAM-1, FN, and other ECM components, leading to continuous inflammation and kidney damage. Different concentrations of TFN can reduce the expression of TGF-β1, Smad-2, α-SMA, CTGF, I collagen, NF-KB, and FN.

Fatty acid biosynthetic pathways include palmitic acid, oleic acid, and arachidonic acid. Oleic acid is a monounsaturated w-9 fatty acid, which is the most widely distributed fatty acid with the highest fat content in nature. Interstitial cell apoptosis can be induced by the production of ceramide. Studies have shown that palmitic acid can induce apoptosis of renal tubular epithelial cells, upregulate cPLA2, produce free fatty acids and lysophosphatidylcholine and other biologically active components such as arachidonic acid, and participate in the occurrence and development of tissue fibrosis (Mu et al., 2001; Allison, 2015). Arachidonic acid can be hydrolyzed to release and generate various active substances, such as 20-hydroxyeicosatetraenoic acid. As the lead compound synthesized by prostaglandin F2a, arachidonic acid metabolism plays an important role in the inflammatory reaction process and is related to kidney disease. It can be used for platelet depolymerization and vasodilation, reducing hypoxia in kidney tissue, improving the body's ability to resist hypoxia, scavenging free radicals, improving hemodynamics, regulating hyperlipidemia, and improving hypercoagulability (Skibba et al., 2017). PCs are also important metabolites in lipid metabolism related to the pathogenesis of RF. In this study, two types of lipid metabolism were discovered, that is, glycerophospholipid and sphingolipid metabolism (Ferro et al., 2018). The decreased LysoPC (17:0) and LysoPC (15:0) that could induce RF by causing disorder of glycerophospholipid metabolism, along with the increased LysoPC (16:1 (9Z)), were observed in the model group. The elevated levels of SM (d18:1/16:0) and decreased sphinganine and galabiosylceramide were observed in the model group. Sphingosine can promote cell growth, adhesion, migration, and death. SM (d18: 1/16: 0) has the activity of hydrolysis to ceramide, which significantly promotes the formation of tissue damage and fibrosis (Dincer et al., 2019; Gai et al., 2019). Citric acid and isocitric acid are intermediates of the tricarboxylic acid cycle (Biasioli et al., 1987). According to these reports, citric acid can significantly shorten the recovery time of urine and stay in the ICU, improve renal function indicators, blood biochemical indicators, and inflammation indicators, maintain the stability of the internal environment, and reduce the risk of bleeding. This study found that the content of citric acid in the model group was decreased, resulting in a decrease in the synthesis of its downstream product isocitrate. After TFN treatment, the content of the two has been called back. Glutamine, as a coding amino acid in protein synthesis is a nonessential amino acids in mammals, can be converted from glucose in the body. It is the most abundant amino acid in the human body and participates in more metabolic processes than any other amino acid. Glutamine reduces kidney damage associated with renal ischemia/reperfusion nitrosation and oxidative stress, attenuates the decrease in Cox-2 expression after I/R, and prevents the increase in AT-1 expression. The reduction of glutamine content in model rats is related to decreased immune system function, imbalance of metabolic nitrogen, blocked protein synthesis, and release of inflammatory factors in the pathogenesis of renal fibrosis (Alba-Loureiro et al., 2010; Li et al., 2019). The abnormal levels of citric acid, isocitric acid, and glutamine lead to the disturbed citrate cycle (TCA cycle), alanine, aspartate, glutamate metabolism, and glyoxylate and dicarboxylate metabolism in RF animals. Ornithine can be used for nutritional supplementation, acute and chronic liver diseases such as cirrhosis, fatty liver, hyperammonemia caused by hepatitis, and central nervous system symptoms. The ornithine cycle converts the more toxic ammonia produced by protein metabolism in the body into the less toxic urea, which is excreted from the body (Montaguth et al., 2019). Cyclic GMP, an important inhibitor of RF synthesized by guanylate cyclase stimulated by nitric oxide or natriuretic peptide, has pleiotropic regulatory functions in the kidney (Lieb et al., 2009). TFN could downregulate the serum content of ornithine and uric acid and upregulate the serum content of glutamine, picolinic acid, and cyclic GMP by adjusting arginine and proline metabolism, arginine biosynthesis, and purine metabolism.

Compared with the UUO + TFN low group, UUO + TFN high group can further affect five metabolic pathways, including PA, tyrosine and tryptophan biosynthesis, PA metabolism, terpenoid backbone biosynthesis, pyrimidine metabolism, and tryptophan metabolism to achieve RF treatment. PA, an essential amino acid in the human body, is involved in the formation of various protein components but cannot be synthesized in the human body. Under normal circumstances, about 50% of the PA consumed is used to synthesize proteins of various components, and the rest is converted to tyrosine under the action of PA hydroxylase and then converted into dopamine, epinephrine, norepinephrine, and melanin. When PA hydroxylase is lacking, these metabolites reach abnormally high levels and accumulate in tissues, plasma, and cerebrospinal fluid, which are excreted in large quantities from the urine (Alkaitis and Ackerman, 2016). PA level in blood sample was decreased after TFN treatment involved in PA, tyrosine, and tryptophan biosynthesis and PA metabolism. 3-Hydroxyanthranilic acid (3-HAA) is a tryptophan metabolite with anti-inflammatory activity, in which the immunoregulatory molecular mechanism of 3-HAA on macrophages is inhibiting the production of inflammatory mediators and reducing NF-κB activity. The results show that 3-HAA has an immunomodulatory effect, which may be due to the inhibition of PI3K/Akt/mTOR and NF-κB activation, thereby reducing the production of proinflammatory mediators in tryptophan metabolism (Krause et al., 2011). The activation of the MAP kinase pathway and MC proliferation by mevalonic acid depletion and might have protective effects by inhibiting IGF-1-mediated MC proliferation. TFN regulates terpenoid backbone biosynthesis activity in RF rats to lower the level of mevalonic acid-5P (Shibata et al., 2009). Disturbances of cerebral purine and pyrimidine metabolism have existed in young children with chronic renal failure (Gerrits et al., 1991). In our present study, serum levels of glutamine and deoxyuridine were remarkably increased in the RF model group. It could be concluded that TFN could reregulate the expression of the above metabolites and perturbed pathways, suggesting the renal-protective effects on UUO-induced RF rats.

## Conclusion

In this study, our work indicated that TFN possesses an important protective effect in an UUO-induced rat RF model by relieving the perturbed level of 27 metabolites associated with the vital pathways. The therapeutic effects of TFN were confirmed in UUO-induced animal model, which was linked with the delayed pathological development and reversal of the perturbed metabolic biomarkers and pathways at the molecule level. Metabolomics, a valuable and promising strategy, is conducive to better understand natural product pretreatment mechanism facing disease and provide novel thought to develop a therapeutic agent in RF. The study is conducive to us for understanding the pathogenesis of RF and bringing about an emerging potential natural antifibrosis agent.

## Data Availability Statement

The datasets presented in this study can be found in online repositories. The names of the repository/repositories and accession number(s) can be found in the article/Supplementary Material.

## Ethics Statement

The animal study was reviewed and approved by the Guide for the Care and Use of Guilin Medical University.

## Author Contributions

Y-SW and ZY designed the experiments; LR, H-NG, JY, X-YG, Y-SW, and ZY performed the experiment; LR, H-NG, JY, and X-YG analyzed the data; LR wrote the paper. All the authors read and approved the final manuscript.

## Conflict of Interest

The authors declare that the research was conducted in the absence of any commercial or financial relationships that could be construed as a potential conflict of interest.

## Abbreviations

TFN, taxifolin; UUO, unilateral ureteral obstruction; CKD, chronic kidney disease; RF, renal fibrosis; ECM, extracellular matrix; NMR, nuclear magnetic resonance; GC-MS, gas chromatography coupled with mass spectrometry; CE-MS, capillary electrophoresis coupled with mass spectrometry; MS, mass spectrometry; CRF, chronic renal failure; TGF-β1, transforming growth factor-β1; Smad-2, small mothers against decapentaplegic; α-SMA, α-smooth muscle actin; CTGF, connective tissue growth factor; NF-KB, nuclear factor-kappa B; PCA, principal component analysis; OPLS-DA, orthogonal partial least squares discriminant analysis; VIP, variable weight value.

## References

[B1] Alba-Loureiro T. C. Ribeiro R. F. Zorn T. M. Lagranha C. J. (2010). Effects of glutamine supplementation on kidney of diabetic rat. Amino Acids. 38 (4), 1021–1030. 10.1007/s00726-009-0310-3 19533301

[B2] Alkaitis M. S. Ackerman H. C. (2016). Tetrahydrobiopterin supplementation improves phenylalanine metabolism in a murine model of severe malaria. ACS Infect. Dis. 2 (11), 827–838. 10.1021/acsinfecdis.6b00124 27641435 PMC6289270

[B3] Allison S. J. (2015). Fibrosis: dysfunctional fatty acid oxidation in renal fibrosis. Nat. Rev. Nephrol. 11 (2), 64 10.1038/nrneph.2014.244 25536395

[B4] Biasioli S. Feriani M. Bigi L. Dell’Aquila R. Bragantini L. Chiaramonte S. (1987). Tricarboxylic acid cycle intermediates in chronic renal failure. Nephrol. Dial. Transplant. 2 (5), 313–315. 3122107

[B5] Boor P. Ostendorf T. Floege J. (2010). Renal fibrosis: novel insights into mechanisms and therapeutic targets. Nat. Rev. Nephrol. 6 (11), 643–656. 10.1038/nrneph.2010.120 20838416

[B6] Casadei L. Valerio M. Manetti C. (2018). Metabolomics: challenges and opportunities in systems biology studies. Methods Mol. Biol. 1702, 327–336. 10.1007/978-1-4939-7456-6_16 29119513

[B7] Casaschi A. Rubio B. K. Maiyoh G. K. Theriault A. G. (2004). Inhibitory activity of diacylglycerolacyltransferase (DGAT) and microsomal triglyceride transfer protein(MTP) by the flavonoid, taxifolin, in HepG2 cells: potential role in the regulation of apolipoprotein B secretion. Atherosclerosis. 176, 247–253. 10.1016/j.atherosclerosis.2004.05.020 15380446

[B8] Chen J. Li D. (2018). Telbivudine attenuates UUO-induced renal fibrosis via TGF-β/Smad and NF-κBsignaling. IntImmunopharmacol. 55, 1–88. 10.1016/j.intimp.2017.11.043 29207359

[B9] Chen P. S. Li Y. P. Ni H. F. (2019). Morphology and evaluation of renal fibrosis. Adv. Exp. Med. Biol. 1165, 17–36. 10.1007/978-981-13-8871-2_2 31399959

[B10] Dincer N. Dagel T. Afsar B. Covic A. Ortiz A. Kanbay M. (2019). The effect of chronic kidney disease on lipid metabolism. Int. Urol. Nephrol. 51 (2), 265–277. 10.1007/s11255-018-2047-y 30519980

[B11] Donnahoo K. K. Shames B. D. Harken A. H. Meldrum D. R. (1999). Reviewarticle: the role of tumor necrosis factor in renal ischemia-reperfusion injury. J. Urol. 162 (1), 196–203. 10.1097/00005392-199907000-00068 10379787

[B12] Fang H. Zhang A. H. Sun H. Yu J. B. Wang L. Wang X. J. (2019). High-throughput metabolomics screen coupled with multivariate statistical analysis identifies therapeutic targets in alcoholic liver disease rats using liquid chromatography-mass spectrometry. J Chromatogr B AnalytTechnol Biomed Life Sci. 1109, 112–120. 10.1016/j.jchromb.2019.01.017 30743140

[B13] Fang J. Wang W. Sun S. Wang Y. Li Q. Lu X. (2016). Metabolomics study of renal fibrosis and intervention effects of total aglycone extracts of Scutellariabaicalensis in unilateral ureteral obstruction rats. J. Ethnopharmacol. 192, 20–29. 10.1016/j.jep.2016.06.014 27286917

[B14] Ferro C. J. Mark P. B. Kanbay M. Sarafidis P. Heine G. H. Rossignol P. (2018). Lipid management in patients with chronic kidney disease. Nat. Rev. Nephrol. 14 (12), 727–749. 10.1038/s41581-018-0072-9 30361677

[B15] Gai Z. Wang T. Visentin M. Kullak-Ublick G. A. Fu X. Wang Z. (2019). Lipid accumulation and chronic kidney disease. Nutrients. 11 (4), 722 10.3390/nu11040722 PMC6520701 30925738

[B16] Gerrits G. P. Monnens L. A. De Abreu R. A. Schröder C. H. Trijbels J. M. Gabreëls F. J. (1991). Disturbances of cerebral purine and pyrimidine metabolism in young children with chronic renal failure. Nephron. 58 (3), 310–314. 10.1159/000186442 1896096

[B17] Gocer H. Topal F. Topal M. Küçük M. Teke D. Gülçin İ. (2016). Acetylcholinesterase and carbonic anhydrase isoenzymes I and II inhibition profiles of taxifolin. J. Enzym. Inhib. Med. Chem. 31, 441–447. 10.3109/14756366.2015.1036051 25893707

[B18] Gowda G. A. Djukovic D. (2014). Overview of mass spectrometry-based metabolomics: opportunities and challenges. Methods Mol. Biol. 1198, 3–12. 10.1007/978-1-4939-1258-2_1 25270919 PMC4336784

[B19] Guo H. Zhang X. Cui Y. Zhou H. Xu D. Shan T. (2015). Taxifolin protects against cardiac hypertrophy and fibrosis during biomechanical stress of pressure overload. Toxicol. Appl. Pharmacol. 287, 168–177. 10.1016/j.taap.2015.06.002 26051872

[B20] Hosten A. O. (1990). “BUN and creatinine,” in Clinical methods: the history, physical, and laboratory examinations. Editors Walker H. K. Hall W. D. Hurst J. W. 3rd ed. (Boston: Butterworths) 21250045

[B21] Hu X. Sang Y. Yang M. Chen X. Tang W. (2018). Prevalence of chronic kidney disease-associated pruritus among adult dialysis patients: a meta-analysis of cross-sectional studies. Medicine (Baltim.). 97 (21), e10633 10.1097/MD.0000000000010633 PMC6392722 29794739

[B22] In S. Yook N. Kim J. H. Shin M. Tak S. Jeon J. H. (2019). Enhancement of exfoliating efficacy of L-carnitine with ion-pair method monitored by nuclear magnetic resonance spectroscopy. Sci. Rep. 9, 13507 10.1038/s41598-019-49818-2 31534155 PMC6751292

[B23] Inoue T. Saito S. Tanaka M. Yamakage H. Kusakabe T. Shimatsu A. (2019). Pleiotropic neuroprotective effects of taxifolin in cerebral amyloid angiopathy. Proc. Natl. Acad. Sci. U S A. 116 (20), 10031–10038. 10.1073/pnas.1901659116 31036637 PMC6525485

[B24] Jenkins J. Brodsky S. V. Satoskar A. A. Nadasdy G. Nadasdy T. (2011). The relevance of periglomerular fibrosis in the evaluation of routine needle core renal biopsies. Arch. Pathol. Lab Med. 135 (1), 117–122. 10.1043/2009-0484-OAR1.1 21204717

[B25] Kakitapalli Y. Ampolu J. Madasu S. D. Sai Kumar M. L. S. (2020). Detailed review of chronic kidney disease. Kidney Dis. 6 (2), 85–91. 10.1159/000504622 PMC7154282 32309290

[B26] Krause D. Suh H. S. Tarassishin L. Cui Q. L. Durafourt B. A. Choi N. (2011). The tryptophan metabolite 3-hydroxyanthranilic acid plays anti-inflammatory and neuroprotective roles during inflammation: role of hemeoxygenase-1. Am. J. Pathol. 179 (3), 1360–1372. 10.1016/j.ajpath.2011.05.048 21855684 PMC3157215

[B27] Lai C. Q. Smith C. E. Parnell L. D. Lee Y.-C. Corella D. Hopkins P. (2018). Epigenomics and metabolomics reveal the mechanism of the APOA2-saturated fat intake interaction affecting obesity. Am. J. Clin. Nutr. 108 (1), 188–200. 10.1093/ajcn/nqy081 29901700 PMC6454512

[B28] Li X. N. Zhang A. Wang M. Sun H. Liu Z. Qiu S. (2017). Screening the active compounds of Phellodendri Amurensis cortex for treating prostate cancer by high-throughput chinmedomics. Sci. Rep. 7, 46234 10.1038/srep46234 28383015 PMC5382783

[B29] Li Y. F. Qiu S. Gao L. J. Zhang A.-H. (2018). Metabolomic estimation of the diagnosis of hepatocellular carcinoma based on ultrahigh performance liquid chromatography coupled with time-of-flight mass spectrometry. RSC Adv. 8 (17), 9375–9382. 10.1039/c7ra13616a PMC9078651 35541871

[B30] Li X. L. Xiao H. T. Li Y. C. Li Y. G. Zhang J. Feng K. (2019). Effects of citric acid on patients with severe burn complicated with acute renal injury treated by continuous renal replacement therapy. Zhonghua Shao Shang ZaZhi. 35 (8), 568–573. 10.3760/cma.j.issn.1009-2587.2019.08.003 31474035

[B31] Li S. Xu Y. Guo W. Chen F. Zhang C. Tan H. Y. (2020). The impacts of herbal medicines and natural products on regulating the hepatic lipid metabolism. Front. Pharmacol. 11, 351 10.3389/fphar.2020.00351 32265720 PMC7105674

[B32] Lieb D. C. Kemp B. A. Howell N. L. Gildea J. J. Carey R. M. (2009). Reinforcing feedback loop of renal cyclic Gmp and interstitial hydrostatic pressure in pressure-natriuresis. Hypertension. 54 (6), 1278–1283. 10.1161/HYPERTENSIONAHA.109.131995 19841292 PMC3057527

[B33] Liu Y. (2011). Cellular and molecular mechanisms of renal fibrosis. Nat. Rev. Nephrol. 7 (12), 684–696. 10.1038/nrneph.2011.149 22009250 PMC4520424

[B34] López-Hernández F. J. López-Novoa J. M. (2012). Role of TGF-β in chronic kidney disease: an integration of tubular,glomerular and vascular effects. Cell Tissue Res. 347 (1), 141 10.1007/s00441-011-1275-6 22105921

[B35] Manigandan K. Manimaran D. Jayaraj R. L. Elangovan N. Dhivya V. Kaphle A. (2015). Taxifolin curbs NF-kappaB-mediated Wnt/beta-catenin signaling via up-regulating Nrf2 pathway in experimental colon carcinogenesis. Biochimie. 119, 103–112. 10.1016/j.biochi.2015.10.014 26482805

[B36] Montaguth O. E. T. Bervoets I. Peeters E. Charlier D. (2019). Competitive repression of the art PIQM operon for arginine and ornithine transport by arginine repressor and leucine-responsive regulatory protein in *Escherichia coli* . Front. Microbiol. 10, 1563 10.3389/fmicb.2019.01563 31354664 PMC6640053

[B37] Mu Y. M. Yanase T. Nishi Y. Tanaka A. Saito M. Jin C. H. (2001). Saturated FFAs, palmitic acid and stearic acid, induce apoptosis in human granulosa cells. Endocrinology. 142 (8), 3590–3597. 10.1210/endo.142.8.8293 11459807

[B38] Nomani H. Khanmohamadian H. Vaisi-Raygani A. Shakiba E. Tanhapour M. Rahimi Z. (2018). Chemerin rs17173608 and vaspin rs2236242 gene variants on the risk of end stage renal disease (ESRD) and correlation with plasma malondialdehyde (MDA) level. Ren. Fail. 40 (1), 350–356. 10.1080/0886022X.2018.1459698 29644922 PMC6014516

[B39] Oh Y. C. Jeong Y. H. Ha J. H. Cho W. K. Ma J. Y. (2014). Oryeongsan inhibits LPS-induced production of inflammatory mediators via blockade of the NF-kappaB, MAPK pathways and leads to HO-1 induction in macrophage cells. BMC Compl. Alternative Med. 14 (14), 242–245. 10.1186/1472-6882-14-242 PMC4223373 25023125

[B40] Oi N. Chen H. Kim M. O. Lubet R. A. Bode A. M. Dong Z. (2012). Taxifolin suppresses UV-induced skin carcinogenesis by targeting EGFR and PI3K. Canc. Prev. Res. 5, 1103–1114. 10.1158/1940-6207.CAPR-11-0397 PMC3435475 22805054

[B41] Qin W. Chung A. C. K. Huang X. R. Meng X. M. Hui D. S. Yu C. M. (2011). TGF-b/Smad3 signaling promotes renal fibrosis by inhibiting miR-29. J. Am. Soc. Nephrol. 22 (8), 1462–1474. 10.1681/ASN.2010121308 21784902 PMC3148701

[B42] Rayego-Mateos S. Valdivielso J. M. (2020). New therapeutic targets in chronic kidney disease progression and renal fibrosis. Expert Opin. Ther. Targets. 27 (7), 655–670. 10.1080/14728222.2020.1762173 32338087

[B43] Sachan R. Kundu A. Dey P. Son J. Y. Kim K. S. Lee D. E. (2020). Dendropanax morbifera protects against renal fibrosis in streptozotocin-induced diabetic rats. Antioxidants. 9 (1), E84 10.3390/antiox9010084 31963869 PMC7023400

[B44] Schauss A. G. Tselyico S. S. Kuznetsova V. A. Yegorova I. (2015). Toxicological and genotoxicity assessment of a dihydroquercetin-rich dahurian larch tree (LarixgmeliniiRupr) extract (lavitol). Int. J. Toxicol. 34 (2), 162–181. 10.1177/1091581815576975 25850419

[B45] Schrimpe-Rutledge A. C. Codreanu S. G. Sherrod S. D. McLean J. A. (2016). Untargeted metabolomics strategies-challenges and emerging directions. J. Am. Soc. Mass Spectrom. 27 (12), 1897–1905. 10.1007/s13361-016-1469-y 27624161 PMC5110944

[B46] Shen Y. L. Jiang Y. P. Li X. Q. Wang S. J. Ma M. H. Zhang C. Y. (2019). ErHuang formula improves renal fibrosis in diabetic nephropathy rats by inhibiting CXCL6/JAK/STAT3 signaling pathway. Front. Pharmacol. 10, 1596 10.3389/fphar.2019.01596 32038260 PMC6993046

[B47] Shibata T. Tamura M. Kabashima N. Serino R. Tokunaga M. Matsumoto M. (2009). Fluvastatin attenuates IGF-1-induced ERK1/2 activation and cell proliferation by mevalonic acid depletion in human mesangial cells. Life Sci. 84 (21–22), 725–731. 10.1016/j.lfs.2009.02.022 19254730

[B48] Skibba M. Hye Khan M. A. Kolb L. L. Yeboah M. M. Falck J. R. Amaradhi R. (2017). Epoxyeicosatrienoic acid analog decreases renal fibrosis by reducing epithelial-to-mesenchymal transition. Front. Pharmacol. 8, 406 10.3389/fphar.2017.00406 28713267 PMC5491687

[B49] Slimestad R. Fossen T. Vågen I. M. (2007). Onions: a source of unique dietary flavonoids. J. Agric. Food Chem. 55 (25), 10067–10080. 10.1021/jf0712503 17997520

[B50] Son D. Kojima I. Inagi R. Matsumoto M. Fujita T. Nangaku M. (2008). Chronic hypoxia aggravates renal injury via suppression of Cu/Zn-SOD: a proteomic analysis. Am. J. Physiol. Ren. Physiol. 294 (1), F62–F72. 10.1152/ajprenal.00113.2007 17959751

[B51] Stevens V. A. Saad S. Chen X. M. Pollock C. A. (2007). The interdependence of EGF-R and SGK-1 in fibronectin expression in primary kidney cortical fibroblast cells. Int. J. Biochem. Cell Biol. 39 (5), 1047–1054. 10.1016/j.biocel.2007.02.013 17382577

[B52] Suh S. H. Lee K. E. Kim I. J. Kim O. Kim C. S. Choi J. S. (2015). Alpha-lipoic acid attenuates lipopolysaccharide-induced kidney injury. Clin. Exp. Nephrol. 19 (1), 82–91. 10.1007/s10157-014-0960-7 24643788

[B53] Sun H. Zhang A. Wang X. (2012). Potential role of metabolomic approaches for Chinese medicine syndromes and herbal medicine. Phytother Res. 26 (10), 1466–1471. 10.1002/ptr.4613 22422429

[B54] Sun X. Chen R. C. Yang Z. H. Sun G. B. Wang M. Ma X. J. (2014). Taxifolin prevents diabetic cardiomyopathy *in vivo* and *in vitro* by inhibition of oxidative stress and cell apoptosis. Food Chem. Toxicol. 63, 221–232. 10.1016/j.fct.2013.11.013 24269735

[B55] Sun H. Zhang A. H. Liu S. B. Qiu S. Li X.-N. Zhang T.-L. (2018a). Cell metabolomics identify regulatory pathways and targets of magnoline against prostate cancer. J Chromatogr B AnalytTechnol Biomed Life Sci. 1102–1103, 143–151. 10.1016/j.jchromb.2018.10.017 30391728

[B56] Sun H. Zhang A. H. Song Q. Fang H. Liu X.-Y. Su J. (2018b). Functional metabolomics discover pentose and glucuronateinterconversion pathways as promising targets for Yang Huang syndrome treatment with Yinchenhao Tang. RSC Adv. 8, 36831–36839. 10.1039/c8ra06553e PMC9089300 35558940

[B57] Telenti A. (2018). Integrating metabolomics with genomics. Pharmacogenomics. 19 (18), 1377–1381. 10.2217/pgs-2018-0155 30398072

[B58] Tokuoka S. M. Kita Y. Shimizu T. Oda Y. (2019). Isobaric mass tagging and triple quadrupole mass spectrometry to determine lipid biomarker candidates for Alzheimer’s disease. PLoS One. 14 (12), e0226073 10.1371/journal.pone.0226073 31821352 PMC6903722

[B59] Topal F. Nar M. Gocer H. Kalin P. Kocyigit U. M. Gülçin İ. (2016). Antioxidant activity of taxifolin: an activity–structure relationship. J. Enzym. Inhib. Med. Chem. 31 (4), 674–683. 10.3109/14756366.2015.1057723 26147349

[B60] Vasko R. (2016). Peroxisomes and kidney injury. Antioxidants Redox Signal. 25, 217–231. 10.1089/ars.2016.6666 PMC4964762 26972522

[B61] Vladimirov Y. A. Proskurnina E. V. Demin E. M. Matveeva N. S. Lubitskiy O. B. Novikov A. A. (2009). Dihydroquercetin (taxifolin) and other flavonoids as inhibitors of free radical formation at key stages of apoptosis. Biochemistry Mosc. 74, 301–307. 10.1134/s0006297909030092 19364325

[B62] Voulgari C. Papadogiannis D. Tentolouris N. (2010). Diabetic cardiomyopathy: from the pathophysiology of the cardiac myocytes to current diagnosis and management strategies. Vasc. Health Risk Manag. 6, 883–903. 10.2147/VHRM.S11681 21057575 PMC2964943

[B63] Wang H. Yan G. Zhang A. Li Y. Wang Y. Sun H. (2013). Rapid discovery and global characterization of chemical constituents and rats metabolites of Phellodendriamurensis cortex by ultra-performance liquid chromatography-electrospray ionization/quadrupole-time-of-flight mass spectrometry coupled with pattern recognition approach. Analyst. 138 (11), 3303–3312. 10.1039/c3an36902a 23608925

[B64] Wang X. Li J. Zhang A. H. (2016). Urine metabolic phenotypes analysis of extrahepaticcholangiocarcinoma disease using ultra-high performance liquid chromatography-mass spectrometry. RSC Adv. 6 (67), 63049–63057. 10.1039/c6ra09430a

[B65] Wang S. Zhou Y. Zhang Y. He X. Zhao X. Zhao H. (2019). Roscovitine attenuates renal interstitial fibrosis in diabetic mice through the TGF-β1/p38 MAPK pathway. Biomed. Pharmacother. 115, 108895 10.1016/j.biopha.2019.108895 31029000

[B66] Wang W. Ma B. L. Xu C. G. Zhou X. J. (2020). Dihydroquercetin protects against renal fibrosis by activating the Nrf2 pathway. Phytomedicine. 69, 153185 10.1016/j.phymed.2020.153185 32120244

[B67] Wu G. D. Compher C. Chen E. Z. Smith S. A. Shah R. D. Bittinger K. (2016). Comparative metabolomics in vegans and omnivores reveal constraints on diet-dependent gut microbiota metabolite production. Gut. 65 (1), 63–72. 10.1136/gutjnl-2014-308209 25431456 PMC4583329

[B68] Xie X. Peng J. Huang K. Huang J. Shen X. Liu P. (2012). Polydatin ameliorates experimental diabetes-induced fibronectin through inhibiting the activation of NF-kappaB signaling pathway in rat glomerular mesangialcells. Mol. Cell. Endocrinol. 362 (1/2), 183–193. 10.1016/j.mce.2012.06.008 22732364

[B69] Xie J. Zhang A. Wang X. (2017). Metabolomic applications in hepatocellular carcinoma: toward the exploration of therapeutics and diagnosis through small molecules. RSC Adv. 7, 17217–17226. 10.1039/c7ra00698e

[B70] Xie J. Zhang A. H. Qiu S. Zhang T. L. Li X. N. Yan G. L. (2019). Identification of the perturbed metabolic pathways associating with prostate cancer cells and anticancer affects of obacunone. J. Proteomics. 206, 103447 10.1016/j.jprot.2019.103447 31326558

[B71] Yin X. N. Wang J. Cui L. F. Fan W. X. (2018). Enhanced glycolysis in the process of renal fibrosis aggravated the development of chronic kidney disease. Eur. Rev. Med. Pharmacol. Sci. 22 (13), 4243–4251. 10.26355/eurrev_201807_15419 30024614

[B72] Zeeh J. (2020). Chronic kidney disease (CKD) in the elderly. MMW - Fortschritte Med. 162 (6), 45–49. 10.1007/s15006-020-0340-z 32248471

[B73] Zeisberg M. Kalluri R. (2004). The role of epithelial-to-mesenchymal transition in renal fibrosis. J. Mol. Med. 82 (3), 175–181. 10.1007/s00109-003-0517-9 14752606

[B74] Zeisberg M. Bonner G. Maeshima Y. Colorado P. Müller G. A. Strutz F. (2002). Renal Fibrosis. collagen composition and assembly regulates epithelial-mesenchymaltransdifferentiation. Am. J. Pathol. 159 (4), 1313–1321. 10.1016/S0002-9440(10)62518-7 PMC1850511 11583959

[B75] Zhang A. Sun H. Qiu S. Wang X. J. (2013a). Metabolomics in noninvasive breast cancer. Clin. Chim. Acta. 424, 3–7. 10.1016/j.cca.2013.05.003 23669185

[B76] Zhang A. Sun H. Xu H. Qiu S. Wang X. (2013b). Cell metabolomics. OMICS A J. Integr. Biol. 17 (10), 495–501. 10.1089/omi.2012.0090 PMC3783970 23988149

[B77] Zhang A. Sun H. Han Y. Yan G. Wang X. (2013c). Urinary metabolic biomarker and pathway study of hepatitis B virus infected patients based on UPLC-MS system. PLoS One. 8 (5), e64381 10.1371/journal.pone.0064381 23696887 PMC3655955

[B78] Zhang A. Sun H. Yan G. Yuan Y. Han Y. Wang X. J. (2014a). Metabolomics study of type 2 diabetes using ultra-performance LC-ESI/quadrupole-TOF high-definition MS coupled with pattern recognition methods. J. Physiol. Biochem. 70 (1), 117–128. 10.1007/s13105-013-0286-z 23975652

[B79] Zhang A. Sun H. Qiu S. Wang X. (2014b). Metabolomics insights into pathophysiological mechanisms of nephrology. Int. Urol. Nephrol. 46 (5), 1025–1030. 10.1007/s11255-013-0600-2 24217804

[B80] Zhang A. Sun H. Yan G. Wang P. Wang X. (2016a). Mass spectrometry-based metabolomics: applications to biomarker and metabolic pathway research. Biomed. Chromatogr. 30 (1), 7–12. 10.1002/bmc.3453 25739660

[B81] Zhang Z. H. Vaziri N. D. Wei F. Cheng X. L. Bai X. Zhao Y. Y. (2016b). An integrated lipidomics and metabolomics reveal nephroprotective effect and biochemical mechanism of Rheum officinale in chronic renal failure. Sci. Rep. 6 (3), 22151 10.1038/srep22151 26903149 PMC4763304

[B82] Zhang Y. Liu P. Li Y. Zhang A.-H. (2017). Exploration of metabolite signatures using high-throughput mass spectrometry coupled with multivariate data analysis. RSC Adv. 7, 6780–6787. 10.1039/c6ra27461g

[B83] Zhang Y. Meng X. M. Huang X. R. Lan H. Y. (2018). The preventive and therapeutic implication for renal fibrosis by targetting TGF-β/Smad3 signaling. Clin. Sci. (Lond.). 132 (13), 1403–1415. 10.1042/CS20180243 29875262

[B84] Zhao M. Chen J. Zhu P. Fujino M. Takahara T. Toyama S. (2015). Dihydroquercetin (DHQ) ameliorated concanavalin A-induced mouse experimental fulminant hepatitis and enhanced HO-1 expression through MAPK/Nrf2 antioxidant pathway in RAW cells. Int. Immunopharm. 28, 938–944. 10.1016/j.intimp.2015.04.032 25916679

[B85] Zhao Y. Lv H. Qiu S. Gao L. Ai H. (2017). Plasma metabolic profiling and novel metabolite biomarkers for diagnosing prostate cancer. RSC Adv. 7 (48), 30060–30069. 10.1039/c7ra04337f

[B86] Zhao Y. Huang W. Wang J. Chen Y. Huang W. Zhu Y. (2018). Taxifolin attenuates diabetic nephropathy in streptozotocin-induced diabetic rats. Am J Transl Res. 10 (4), 1205–1210 29736213 PMC5934579

[B87] Zhao S. Jiang J. T. Li D. Zhu Y.-P. Xia S.-J. Han B.-M. (2019). Maternal exposure to di-n-butyl phthalate promotes Snail1-mediated epithelial-mesenchymal transition of renal tubular epithelial cells via upregulation of TGF-β1 during renal fibrosis in rat offspring. Ecotoxicol. Environ. Saf. 169, 266–272. 10.1016/j.ecoenv.2018.10.073 30453174

[B88] Zheng L. Li Y. Zhou Z. Xiang W. Gong Z. Chen S. (2019). Comparative pharmacokinetics of quercitrin, astragalin, afzelin and taxifolin in plasma after oral administration of Polygonumorientale inflorescence in sham-operated and myocardial ischemia-reperfusion injury rats. Xenobiotica. 13, 1–9. 10.1080/00498254.2019.1700319 31791186

[B89] Zhou C. Liu J. Ge Y. Zhu Y. Zhou L. Xu L. (2020). Remote ischemic preconditioning ameliorates renal fibrosis after ischemia-reperfusion injury via transforming growth factor beta1 (TGF-β1) signalling pathway in rats. Med. Sci. Monit. 26, e919185 10.12659/MSM.919185 32024811 PMC7020740

[B90] Zia K. Siddiqui T. Ali S. Farooq I. Zafar M. S. Khurshid Z. (2019). Nuclear magnetic resonance spectroscopy for medical and dental applications: a comprehensive review. Eur. J. Dermatol. 13 (1), 124–128. 10.1055/s-0039-1688654 PMC6635960 31170770

