# Supplementary material for: Dissecting Efficacy and Metabolic Characteristic Mechanism of Taxifolin on Renal Fibrosis by Multivariate Approach and Ultra-Performance Liquid Chromatography Coupled With Mass Spectrometry-Based Metabolomics Strategy
Source: Front Pharmacol. 2021 Jan 14;11:608511. doi: 10.3389/fphar.2020.608511 (PMC7841412; doi:10.3389/fphar.2020.608511)
Supplement: Supplementary file 1 [file datasheet1.doc]

**Table S1** The distinct metabolites identified in serum samples.

| **No.** | **Mode** | **RT(min)** | **Name** | **Chemical formula** | **m/z** | **VIP value** | **HMDB code** | **Trend in model** | **Low dose of TFN** | **High dose of TFN** |
| --- | --- | --- | --- | --- | --- | --- | --- | --- | --- | --- |
| 1 | M-H | 0.63 | isocitric acid | C6H8O7 | 191.0196 | 2.8342 | HMDB00193 | ↓ | **√** | **√** |
| 2 | M-H | 0.78 | Ornithine | C5H12N2O2 | 131.0816 | 3.7132 | HMDB00214 | ↑ | **√** | **√** |
| 3 | M+H | 0.99 | 3-Hydroxyanthranilic acid | C7H7NO3 | 171.0769 | 1.6790 | HMDB0001476 | ↓ |  | **√** |
| 4 | M+H | 1.35 | 5'-Methylthioadenosine | C11H15N5O3S | 298.0940 | 2.5717 | HMDB0001173 | ↑ |  |  |
| 5 | M+H | 1.84 | Picolinic acid | C6H5NO2 | 124.0394 | 1.7967 | HMDB0002243 | ↓ |  | **√** |
| 6 | M+H | 2.13 | Citric acid | C6H8O7 | 215.0170 | 1.7978 | HMDB0000094 | ↓ | **√** | **√** |
| 7 | M+H | 2.56 | Uric acid | C5H4N4O3 | 169.0360 | 2.9052 | HMDB00289 | ↑ | **√** | **√** |
| 8 | M-H | 2.74 | Asparagine | C4H8N2O3 | 131.0451 | 1.8987 | HMDB33780 | ↓ | **√** | **√** |
| 9 | M+H | 2.56 | Tryptophan | C11H12N2O2 | 205.0995 | 1.6123 | HMDB13609 | ↓ |  | **√** |
| 10 | M-H | 3.27 | Mevalonic acid-5P | C6H13O7P | 273.0376 | 7.8690 | HMDB0001343 | ↑ |  | **√** |
| 11 | M-H | 3.58 | Glutamine | C5H10N2O3 | 145.0613 | 2.8434 | HMDB00641 | ↓ | **√** | **√** |
| 12 | M-H | 3.73 | SM(d18:1/22:0) | C45H91N2O6P | 785.6510 | 1.9432 | HMDB12103 | ↓ | **√** | **√** |
| 13 | M-H | 4.04 | Cysteinylglycine | C5H10N2O3S | 179.0469 | 3.2342 | HMDB00078 | ↓ |  |  |
| 14 | M+H | 4.30 | Kynurenic acid | C10H7NO3 | 212.0280 | 1.6490 | HMDB0000715 | ↓ | **√** | **√** |
| 15 | M-H | 4.77 | Hydroxytyrosol | C8H10O3 | 153.0558 | 5.3948 | HMDB0005784 | ↑ |  | **√** |
| 16 | M+H | 5.14 | Cyclic GMP | C10H12N5O7P | 368.0369 | 3.8718 | HMDB0001314 | ↓ | **√** | **√** |
| 17 | M-H | 5.21 | 20-Hydroxyeicosatetraenoic acid | C20H32O3 | 365.2308 | 1.5912 | HMDB0005998 | ↓ | **√** | **√** |
| 18 | M-H | 5.44 | Deoxyuridine | C9H12N2O5 | 273.0731 | 2.1438 | HMDB0000012 | ↓ |  | **√** |
| 19 | M-H | 6.05 | Pregnenolone sulfate | C21H32O5S | 441.1952 | 1.8200 | HMDB0000774 | ↓ |  |  |
| 20 | M-H | 6.42 | Prostaglandin F2a | C20H34O5 | 399.2419 | 1.9901 | HMDB0001139 | ↑ | **√** | **√** |
| 21 | M+H | 6.87 | Phenylalanine | C9H11NO2 | 166.0848 | 3.1575 | HMDB00159 | ↑ |  | **√** |
| 22 | M+H | 7.22 | Arachidonic acid | C20H32O2 | 327.2304 | 4.5321 | HMDB01043 | ↑ | **√** | **√** |
| 23 | M-H | 7.67 | Dodecanoic acid | C12H24O2 | 199.1685 | 5.2776 | HMDB0000638 | ↓ |  |  |
| 24 | M-H | 7.79 | LysoPC(17:0) | C25H52NO7P | 508.3428 | 2.0464 | HMDB12108 | ↓ |  | **√** |
| 25 | M+H | 8.04 | LysoPC(15:0) | C23H48NO7P | 482.3245 | 2.4888 | HMDB10381 | ↓ | **√** | **√** |
| 26 | M-H | 8.57 | Palmitoleic acid | C16H30O2 | 253.2162 | 2.5998 | HMDB03229 | ↑ | **√** | **√** |
| 27 | M+H | 8.67 | SM（D18:0/16:1） | C39H79N2O6P | 703.5770 | 3.3967 | HMDB0013464 | ↑ |  | **√** |
| 28 | M+H | 8.95 | Dityrosine | C18H20N2O6 | 383.1278 | 2.0208 | HMDB0006045 | ↑ |  |  |
| 29 | M+H | 8.62 | Galabiosylceramide | C46H87NO13 | 862.6232 | 3.5034 | HMDB04833 | ↓ |  | **√** |
| 30 | M-H | 9.08 | LysoPC(16:1(9Z)) | C24H48NO7P | 492.3072 | 4.6609 | HMDB10383 | ↑ |  | **√** |
| 31 | M-H | 9.19 | Oleic acid | C18H34O2 | 281.2482 | 2.4067 | HMDB00207 | ↓ | **√** | **√** |
| 32 | M+H | 10.11 | Sphinganine | C18H39NO2 | 302.3058 | 3.1310 | HMDB00269 | ↓ | **√** | **√** |


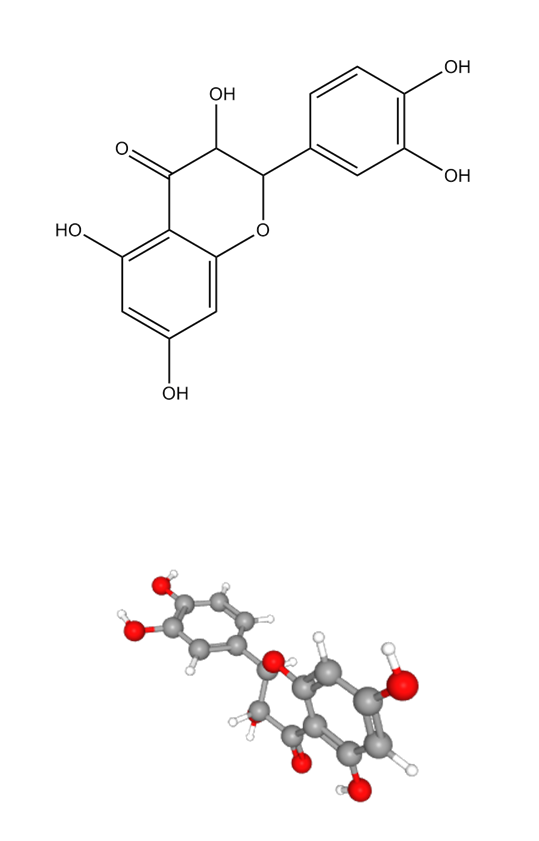


**Figure S1** The 2D structure and 3D interactive chemical structure model of Taxifolin


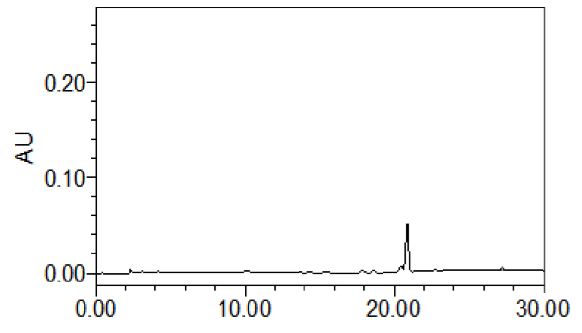


**Figure S2**. HPLC Chromatographic conditions

Chromatographic column: ODS-2 C18 column (250× 4.6mm, 5μm); detection wavelength: 227nm; mobile phase: acetonitrile (A)-water (B). Gradient elution, the elution procedure is: 0～17min(46%A); 17～18min(46% A～100% A); 18～29min(100%A); 29～30min(100%A～46% A).


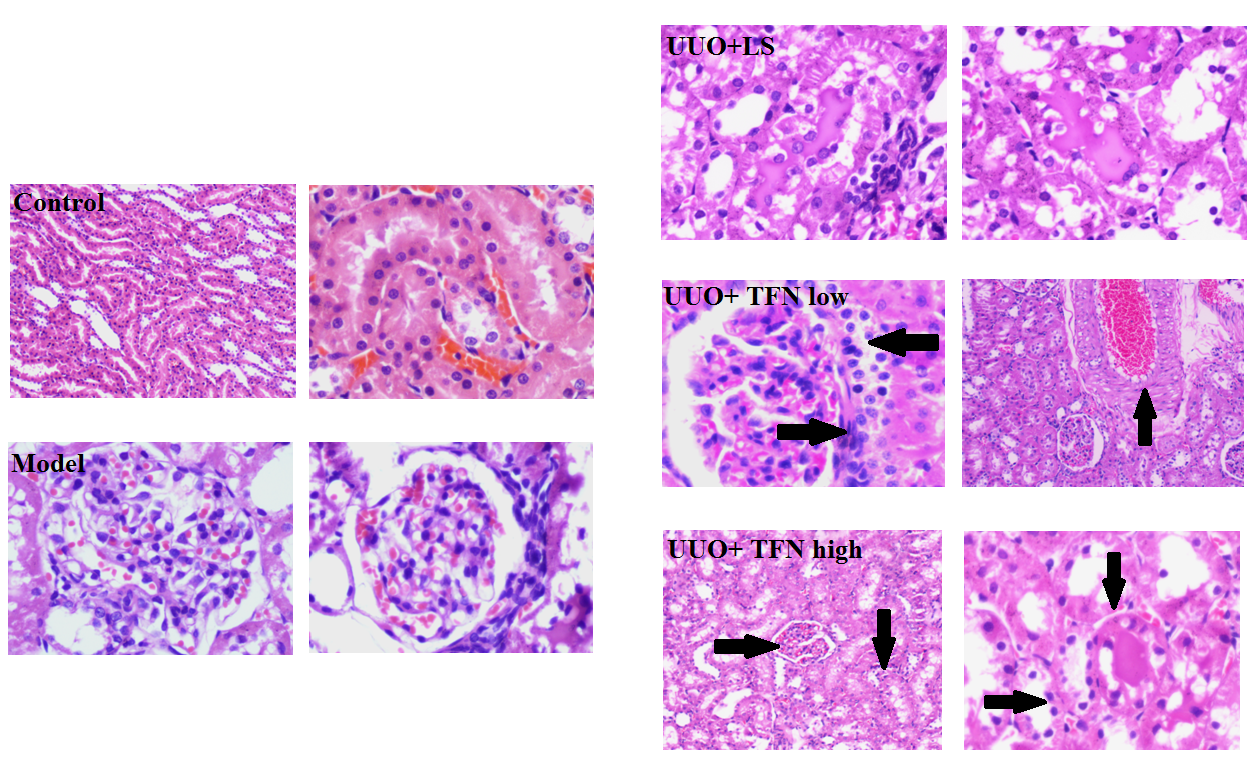


**Figure S3** Hematoxylin and eosin (H&E) staining of the kidney tissue.


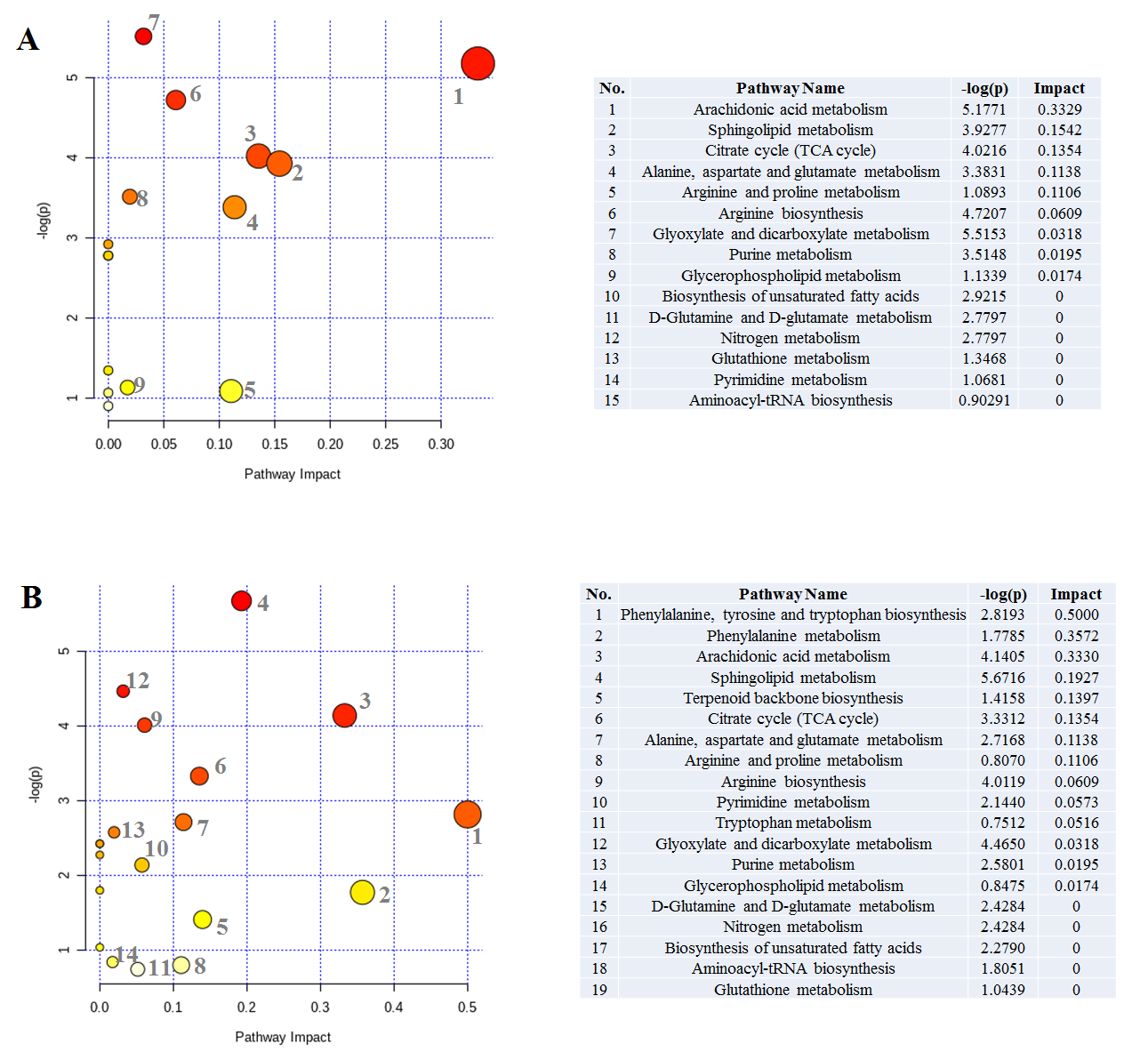


**Figure S4** The metabolic pathways associated with potential biomarkers in rat serum after TFN administration, and -log(p) values and impact value of related metabolic pathways. Note: (A) UUO+TFN low group; (B) UUO+TFN high group.
